# Gestational age and hospital admissions during childhood: population based, record linkage study in England (TIGAR study)

**DOI:** 10.1136/bmj.m4075

**Published:** 2020-11-25

**Authors:** Victoria Coathup, Elaine Boyle, Claire Carson, Samantha Johnson, Jennifer J Kurinzcuk, Alison Macfarlane, Stavros Petrou, Oliver Rivero-Arias, Maria A Quigley

**Affiliations:** 1 National Perinatal Epidemiology Unit, Nuffield Department of Population Health, University of Oxford, Old Road Campus, Headington, Oxford OX3 7FL, UK; 2 Department of Health Sciences, University of Leicester, Leicester, UK; 3 Department of Health Sciences, City University, London, UK; 4 Nuffield Department of Primary Care Health, University of Oxford, UK

## Abstract

**Objective:**

To examine the association between gestational age at birth and hospital admissions to age 10 years and how admission rates change throughout childhood.

**Design:**

Population based, record linkage, cohort study in England.

**Setting:**

NHS hospitals in England, United Kingdom.

**Participants:**

1 018 136 live, singleton births in NHS hospitals in England between January 2005 and December 2006.

**Main outcome measures:**

Primary outcome was all inpatient hospital admissions from birth to age 10, death, or study end (March 2015); secondary outcome was the main cause of admission, which was defined as the World Health Organization’s first international classification of diseases, version 10 (ICD-10) code within each hospital admission record.

**Results:**

1 315 338 admissions occurred between 1 January 2005 and 31 March 2015, and 831 729 (63%) were emergency admissions. 525 039 (52%) of 1 018 136 children were admitted to hospital at least once during the study period. Hospital admissions during childhood were strongly associated with gestational age at birth (<28, 28-29, 30-31, 32, 33, 34, 35, 36, 37, 38, 39, 40, 41, and 42 weeks). In comparison with children born at full term (40 weeks’ gestation), those born extremely preterm (<28 weeks) had the highest rate of hospital admission throughout childhood (adjusted rate ratio 4.92, 95% confidence interval 4.58 to 5.30). Even children born at 38 weeks had a higher rate of hospital admission throughout childhood (1.19, 1.16 to 1.22). The association between gestational age and hospital admission decreased with increasing age (interaction P<0.001). Children born earlier than 28 weeks had an adjusted rate ratio of 6.34 (95% confidence interval 5.80 to 6.85) at age less than 1 year, declining to 3.28 (2.82 to 3.82) at ages 7-10, in comparison with those born full term; whereas in children born at 38 weeks, the adjusted rate ratios were 1.29 (1.27 to 1.31) and 1.16 (1.13 to 1.19), during infancy and ages 7-10, respectively. Infection was the main cause of excess hospital admissions at all ages, but particularly during infancy. Respiratory and gastrointestinal conditions also accounted for a large proportion of admissions during the first two years of life.

**Conclusions:**

The association between gestational age and hospital admission rates decreased with age, but an excess risk remained throughout childhood, even among children born at 38 and 39 weeks of gestation. Strategies aimed at the prevention and management of childhood infections should target children born preterm and those born a few weeks early.

## Introduction

The rates of preterm birth (<37 weeks’ gestation) have been increasing since 2000, accounting for approximately 11% of births worldwide in 2014.^1^ Complications arising from preterm birth are now the leading cause of infant mortality in high and middle income countries.^2^ Considerable advances in the care of preterm babies have resulted in higher survival rates,^3^ but they still remain at a higher risk of infant mortality and morbidity than those born at full term (39-41 weeks’ gestation). Studies exploring the long term health consequences of preterm birth indicate that children born preterm are at higher risk of respiratory disease,^4^ ^5^ ^6^ infections,^7^ and neurodevelopmental deficits^8^ throughout childhood. Growing evidence suggests that even babies born at early term (37-38 weeks’ gestation) have a higher risk of complications than those born at full term.^6^ ^7^ ^9^ Therefore, it is important to explore long term health outcomes and to investigate effects by week of gestation at birth.

About 8% of babies born in England and Wales were preterm in 2016.^10^ Although the increased risk of childhood health complications after preterm or early term delivery is well established,^11^ few large, population based studies have investigated the long term health consequences for the full spectrum of gestational age in populations from the United Kingdom. Previous studies have analysed relatively small samples with broad categories of gestational age,^12^ focused on narrow health outcomes,^4^ or analysed data from older cohorts.^13^ Thus results of those studies might not be generalisable to babies born in settings with more advanced medical care or reflect increases in survival rates over the past 30 years for extremely preterm babies.^3^

In addition, evidence suggests that the association between gestational age and hospital admission rates ameliorates over time.^7^ ^9^ ^14^ It remains unclear, however, at what age this begins to happen and how these changes vary by week of gestational age at birth. This information is important to clinicians, policy makers, and parents when assessing future health service use, identifying populations at greatest risk of hospital admission, and developing targeted interventions.

We present findings from the TIGAR study (Tracking the Impact of Gestational Age on Health, Educational and Economic outcomes: a Longitudinal Records Linkage Study), which is a population based, record linkage study using births and hospital admissions throughout childhood in England. The study objectives were to estimate the association between gestational age and hospital admissions from birth up to the age of 10, explore how rates of hospital admission change throughout childhood, and describe the main causes of admission.

## Methods

### Data sources

We conducted a population based, data linkage cohort study in England using data from the Office for National Statistics (ONS) birth registration records linked to death registration records, birth notification records, and Hospital Episode Statistics Admitted Patient Care (HES APC) records.^15^ HES APC contains details of all inpatient admissions to NHS hospitals in England. Accident and emergency department attendances and outpatient appointments are recorded in other HES databases, and were not linked owing to poor quality and incompleteness of the data. The linkage was conducted by NHS Digital in collaboration with the ONS and City, University of London, using deterministic algorithms as part of a previous National Institute for Health Research funded study. A description of the datasets, linkage, and quality assurance has been published elsewhere.^16^ ^17^ Details of additional quality assurance methods used for this study are included in supplementary information, section A. 

### Study population

All live, singleton births occurring in England between 1 January 2005 and 31 December 2006 were included in the study cohort and followed up from birth until 31 March 2015. Children were not eligible for inclusion in the study population if they were not born in an NHS hospital in England (3.2%) or were born to mothers not living in England at the time of birth (0.2%). Further exclusions from the analyses were unlinked records (7.7%) and children whose parents had opted out of their data being used for research (1.3%); poor quality linkages (0.1%); gestational age of less than 23 weeks or more than 42 weeks (0.4%); missing gestational age or birth weight data (0.6%); implausible birth weight for gestational age (that is, birth weight plus or minus two standard deviations from the median week of gestational age, sex, and ethnicity;^18^ 1.3%); died before discharge from the hospital birth admission (0.1%); or if there were concerns about data quality (0.7%; eg, discharge corrected age of < 34 weeks^19^; discharge dates before admission; admission before date of birth; missing admission or discharge dates). Children born after gestation of more than 42 weeks were excluded owing to concerns about the quality of the gestational age data.^20^ ^21^

### Outcomes

The primary outcome was the total number of NHS inpatient hospital admissions during childhood, reported during the following periods: less than 1 year, 1-2 years, 3-4 years, 5-6 years, and 7-10 years. Admissions occurring at least one day after discharge from the birth admission were included in the analysis. We defined “birth admission” as the initial hospital admission relating to the baby’s birth, beginning when the baby is born and ending when the baby is discharged from an NHS hospital. We defined a “subsequent admission” as a period of continued care within an NHS hospital, which ends when the child is discharged. Hospital records with transfer codes (see supplementary information, section C) and two or fewer days between admission and discharge dates were considered part of the same admission. Further details of HES data are described in supplementary information, section B.

Hospital diagnoses were coded in HES using the World Health Organization’s international classification of diseases, 10th revision (ICD-10). Admissions of healthy babies alongside a sick mother were excluded (Z76.3). We used the primary diagnosis code within the first episode of each admission to define the cause of each admission, which were then grouped into the following broad categories^5^: infection; non-infection respiratory; non-infection gastrointestinal; oral cavity; perinatal; congenital anomalies; social issues; mental health; injury; renal and genitourinary; neoplasm; central nervous system; and other (supplementary information, ICD-10 codes).

### Exposures

Gestational age was recorded in the birth notification record by the midwife or doctor attending the birth and estimated using the date of the mother’s last menstrual period, ultrasound dating scan, and the baby’s date of birth. The last menstrual period is calculated, but it is common practice to use the ultrasound scan in early pregnancy to estimate gestational age, as this is generally accepted to be more accurate. No method of assessment was recorded in birth notification, but a dating ultrasound is part of routine antenatal care in the NHS, and almost all women will receive one.^22^ Gestational age was analysed in weeks, using the following categories: less than 28, 28-29, 30-31, 32, 33, 34, 35, 36, 37, 38, 39, 40, 41, and 42. Where numbers were small, gestational ages were grouped (eg, <28, 28-29, and 30-31) to ensure sufficient power to estimate rate ratios. In models with interaction terms, gestational age was grouped using the following categories: less than 28, 28-31, 32-33, 34-36, 37-38, 39-41, and 42. Because gestational age is strongly correlated with birth weight, we investigated fetal growth using sex and gestation specific birth weight centiles and identified those born small for gestational age (defined as a birth weight below the 10th centile for all births).

### Statistical analysis

We performed χ^2^ tests to compare the distribution of all categorical variables in children with and without an admission during childhood. Person years at risk for each child were calculated as the time from discharge from birth admission up to the age of 10 years, death, or the study end (31 March 2015). Crude hospital admission rates per 100 person years were calculated for each category of gestational age within each age band (<1, 1-2, 3-4, 5-6, and 7-10) by dividing the number of subsequent admissions by the person years at risk and multiplying by 100. This calculation was repeated for each cause of admission category. Because rates were so similar for 39-41 and 42 weeks, these categories of gestational age were combined.

Generalised estimating equations with a negative binomial distribution and log link were used to estimate rate ratios for hospital admissions and 95% confidence intervals for each week of gestational age compared with a referent of birth at 40 weeks’ gestation. Generalised estimating equations were also used to account for the correlation between repeated readmissions within and across different age bands (<1, 1-2, 3-4, 5-6, and 7-10), and a negative binomial distribution accounted for the overdispersed data. The models were fitted first to explore the association between gestational age and hospital admission rates, and second to explore whether the association changed over time. For change of association over time, an interaction term was included for gestational age category (<28, 28-31, 32-33, 34-36, 37-38, 39-41, 42) and age at admission in years (<1, 1-2, 3-4, 5-6, and 7-10); this was assessed using the Wald test. Finally, an age stratified analysis was conducted using negative binomial regression models to estimate unadjusted and adjusted rate ratios for admissions by gestational age categories, which were repeated for each of the following age bands (<1, 1-2, 3-4, 5-6, and 7-10). Population attributable fractions were estimated for each band of gestational age as: (proportion of cases exposed) × (RR−1/RR), where RR is the adjusted rate ratio from the generalised estimating equations model.

Models were determined a priori and were adjusted for the following variables based on existing evidence: maternal age at delivery^23^ (<20, 20-24, 25-29, 30-34, 35-39, ≥40); marital status at birth registration^24^ (married, partner, single); area deprivation based on levels of index of multiple deprivation score^25^ ^26^ (for babies born in 2005 and 2006, index of multiple deprivation scores from 2004 and 2007 were used, respectively); child’s ethnicity based on the 2001 census classification (white British, white other, Bangladeshi, Indian, Pakistani, black African, black Caribbean, other); mother’s country of birth^27^ (UK or non-UK born); mode of delivery^28^ (vaginal, caesarean section); parity^9^ ^24^ (nulliparous, parous); month of birth^29^ (January-March, April-June, July-September, October-December), sex^26^ (male, female), and small for gestational age^9^ (yes, no).

We carried out a number of sensitivity analyses to explore the stability of the estimates. Firstly, children considered high risk were excluded (defined as a diagnosis of at least one of the following: malignant neoplasm, a blood disorder, chronic kidney disease, cystic fibrosis, immune dysfunction, or a congenital anomaly; supplementary table S3). Secondly, owing to concerns about the quality of data for the variable parity, a hospital was defined as an unreliable reporter of parity if it reported less than 20% or more than 70% women as nulliparous in 2005 or 2006^16^ (supplementary information, section F). Thirdly, the analysis was restricted to emergency hospital admissions only (supplementary information, section D). Fourthly, the baseline model was further adjusted for labour induction. Fifthly, to account for correlation between children born to the same mother, the analysis was restricted to first born babies during the study period. Sixthly, because very preterm (<32 weeks’ gestation) babies are likely to have long lengths of stay for birth admission, they have a shorter period at risk of subsequent admission and their rates might appear lower. Therefore a z score for length of stay was created for each week of gestational age, then categorised (<1, 0-1, >1) and added to the model in an attempt to adjust for this.^28^ Lastly, the analysis was restricted to children who were not small for gestational age.

All variables had between 0% and 5.8% of missing data, with the exception of labour induction, for which 23% of data were missing. A complete case analysis was conducted. This analysis was deemed a sensible approach because some data were likely to be missing not at random. For example, parity was missing only for unmarried women (supplementary file F provides more detail), and a sensitivity analysis was undertaken to explore its impact. All analyses were conducted using Stata version 14 (College Station, TX).^30^

### Patient and public involvement

The TIGAR study was supported by a patient, parent, and public advisory group, which provided input to different aspects of the study. This group met at the start of the study and gave input into the study protocol and the lay summary of the project.

## Results

**Fig 1:**
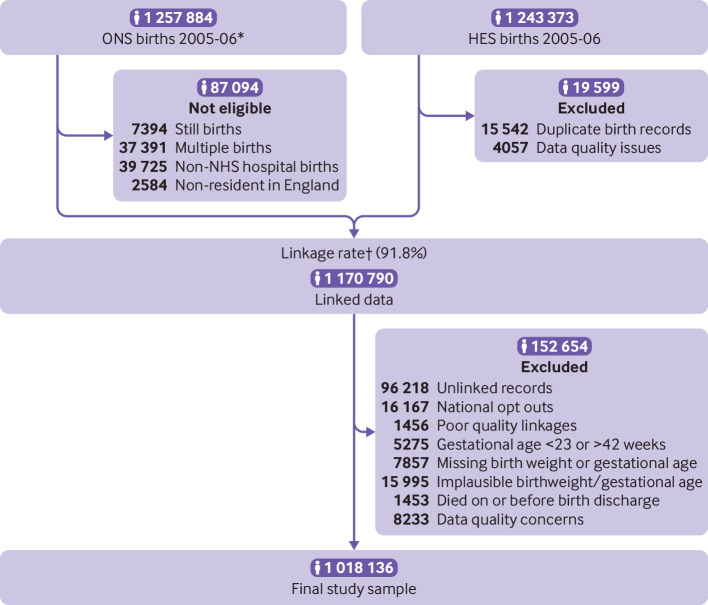
Flowchart of study population. *ONS births comprise routinely linked data from birth registration and birth notification records. †Linkage rate calculated based on eligible births within the master dataset (ONS births). Linkage methods described elsewhere.^17^ HES=Hospital Episode Statistics; ONS=Office for National Statistics

A total of 1 170 790 live, singleton births took place in NHS hospitals, born to mothers living in England between 1 January 2005 and 31 December 2006 (fig 1). After linking and cleaning the datasets, a total of 1 018 136 children remained, with a total of 9 372 105 person years of follow-up and an average of 9.2 years of follow-up per child. A total of 1 315 338 admissions occurred between 1 January 2005 and 31 March 2015, and 831 729 (63%) were emergency admissions. Of the 1 018 136 children, 525 039 (52%) children were admitted on one or more occasions up to the age of 10 (table 1), including 262 606 (26%) who were admitted once, 123 583 (12%) twice, 89 293 (9%) three to four times, and 49 555 (5%) who were admitted on five or more occasions (supplementary table S4).

**Table 1:** Sociodemographic characteristics of sample population (n=1 018 136). Data are number (%) of singleton births

	Total (n=1 018 136)	No admission (n=493 097)	Any admission (n=525 039)
Mother’s age at birth:			
<20	44 486 (4.4)	17 127 (3.5)	27 359 (5.2)
20-24	181 633 (17.8)	76 351 (15.5)	105 282 (20.1)
25-29	253 055 (24.9)	119 340 (24.2)	133 715 (25.5)
30-34	293 741 (28.9)	150 816 (30.6)	142 925 (27.2)
35-39	193 622 (19.0)	102 276 (20.7)	91 346 (17.4)
≥40	51 599 (5.1)	27 187 (5.5)	24 412 (4.6)
Parity:			
Nulliparous	480 616 (47.2)	230 947 (46.8)	249 669 (47.6)
Parous	496 203 (48.7)	241 195 (48.9)	255 008 (48.6)
Missing	41 317 (4.1)	20 955 (4.2)	20 362 (3.9)
Maternal registration status:			
Married	581 160 (57.1)	297 381 (60.3)	283 779 (54.0)
Partner	347 366 (34.1)	158 097 (32.1)	189 269 (36.0)
Single	89 610 (8.8)	37 619 (7.6)	51 991 (9.9)
Mother’s country of birth:			
Non-UK	225 695 (22.2)	121 248 (24.6)	104 447 (19.9)
UK	791 012 (77.7)	371 101 (75.3)	419 911 (80.0)
Missing	1429 (0.1)	748 (0.2)	681 (0.1)
IMD score (groups):			
Group 1 (most deprived)	276 838 (27.2)	120 894 (24.5)	155 944 (29.7)
Group 2	216 006 (21.2)	103 060 (20.9)	112 946 (21.5)
Group 3	180 300 (17.7)	89 733 (18.2)	90 567 (17.2)
Group 4	161 793 (15.9)	82 668 (16.8)	79 125 (15.1)
Group 5 (least deprived)	157 195 (15.4)	83 869 (17.0)	73 326 (14.0)
Missing	26 004 (2.6)	12 873 (2.6)	13 131 (2.5)
Sex:			
Male	521 169 (51.2)	231 013 (46.8)	290 156 (55.3)
Female	496 967 (48.8)	262 084 (53.2)	234 883 (44.7)
Ethnicity (child):			
White British	677 236 (66.5)	299 672 (60.8)	377 564 (71.9)
White other	59 683 (5.9)	32 799 (6.7)	26 884 (5.1)
Bangladeshi	14 546 (1.4)	6669 (1.4)	7877 (1.5)
Indian	27 783 (2.7)	14 517 (2.9)	13 266 (2.5)
Pakistani	41 739 (4.1)	18 583 (3.8)	23 156 (4.4)
Black African	34 571 (3.4)	19 141 (3.9)	15 430 (2.9)
Black Caribbean	12 410 (1.2)	6507 (1.3)	5903 (1.1)
Other	91 570 (9.0)	44 370 (9.0)	47 200 (9.0)
Missing	58 598 (5.8)	50 839 (10.3)	7759 (1.5)

χ^2^ results significant (P<0.001) for all variables. IMD score=index of multiple deprivation score divided into five equal groups (from the lowest, group 1 to the highest, group 5).

Admission on one or more occasion was associated with having a younger, unmarried, UK born mother, living in a more deprived area, being male, white British, preterm, born by caesarean section, small for gestational age, high risk, and having a long birth admission length of stay (table 1 and table 2). Crude admission rates were highest in infancy and for those born preterm. Children born at less than 28 weeks’ gestation had the highest admission rate (253/100 person years), compared with those born at 40 weeks’ gestation (28/100 person years). However, admission rates decreased with increasing chronological age and by ages 7-10, those born at less than 28 weeks’ gestation had a crude admission rate of 26/100 person years, compared with a rate of 7/100 person years for those born at 40 weeks’ gestation (table 3). Even children born a few weeks early had higher admission rates. Being born at 37, 38, and 39 weeks’ gestation was associated with a rate difference of 19, 9, and 3 admissions per 100 person years during infancy, respectively, in comparison with those born at 40 weeks.

**Table 2:** Further birth characteristics of the sample population (n=1 018 136). Data are number (%) of births

Birth characteristics	Total (n=1 018 136)	No admission (n=493 097)	Any admission (n=525 039)
Gestational age (weeks):			
<28	1730 (0.2)	103 (0.0)	1627 (0.3)
28-29	2089 (0.2)	263 (0.1)	1826 (0.3)
30-31	3227 (0.3)	590 (0.1)	2637 (0.5)
32	2656 (0.3)	637 (0.1)	2019 (0.4)
33	4050 (0.4)	1035 (0.2)	3015 (0.6)
34	7292 (0.7)	2225 (0.5)	5067 (1.0)
35	11 663 (1.1)	4051 (0.8)	7612 (1.4)
36	23 346 (2.3)	8822 (1.8)	14 524 (2.8)
37	54 001 (5.3)	22 830 (4.6)	31 171 (5.9)
38	137 926 (13.5)	64 098 (13.0)	73 828 (14.1)
39	231 376 (22.7)	114 208 (23.2)	117 168 (22.3)
40	288 065 (28.3)	145 808 (29.6)	142 257 (27.1)
41	208 757 (20.5)	106 847 (21.7)	101 910 (19.4)
42	41 958 (4.1)	21 580 (4.4)	20 378 (3.9)
Delivery method:			
Vaginal	751 653 (73.8)	368 091 (74.6)	383 562 (73.1)
Caesarean section	222 615 (21.9)	102 853 (20.9)	119 762 (22.8)
Missing	43 868 (4.3)	22 153 (4.5)	21 715 (4.1)
Small for gestational age:			
No	918 419 (90.2)	448 039 (90.9)	470 380 (89.6)
Yes	99 717 (9.8)	45 058 (9.1)	54 659 (10.4)
Labour induction:			
No	626 178 (61.5)	306 105 (62.1)	320 073 (61.0)
Yes	154 851 (15.2)	70 115 (14.2)	84 736 (16.1)
Missing	237 107 (23.3)	116 877 (23.7)	120 230 (22.9)
High risk*:			
No	930 418 (91.4)	476 486 (96.6)	453 932 (86.5)
Yes	87 718 (8.6)	16 611 (3.4)	71 107 (13.5)
Month of birth:			
January-March	236 944 (23.3)	114 296 (23.2)	122 648 (23.4)
April-June	254 016 (24.9)	122 968 (24.9)	131 048 (25.0)
July-September	270 282 (26.5)	131 137 (26.6)	139 145 (26.5)
October-December	256 894 (25.2)	124 696 (25.3)	132 198 (25.2)
Birth admission length of stay:			
<1 week	970 067 (95.3)	480 360 (97.4)	489 707 (93.3)
1-2 weeks	33 589 (3.3)	10 394 (2.1)	23 195 (4.4)
3-4 weeks	6782 (0.7)	1442 (0.3)	5340 (1.0)
1-2 months	4600 (0.5)	701 (0.1)	3899 (0.7)
≥3 months	3098 (0.3)	200 (0.0)	2898 (0.6)

χ^2^ results significant (P<0.001) for all variables, except for month of birth (P=0.13).

* High risk defined as a child with a diagnosis of malignant neoplasm, a blood disorder, chronic kidney disease, cystic fibrosis, immune dysfunction, or a congenital anomaly.

**Table 3:** Descriptive characteristics of hospital admissions by gestational age and age at admission

Gestational age (weeks)	Age at admission																		
	<1 year				1-2 years				3-4 years				5-6 years				7-10 years		
	% ≥1 (R)	PY	Rate		% ≥1 (R)	PY	Rate		% ≥1 (R)	PY	Rate		% ≥1 (R)	PY	Rate		% ≥1 (R)	PY	Rate

<28	68.7 (3146)	1244	253		61.5 (3223)	3413	94		41.0 (1754)	3395	52		30.2 (1174)	3391	35		24.6 (974)	3784	26
28-29	58.8 (2723)	1712	159		47.9 (2398)	4138	58		32.8 (1470)	4130	36		26.1 (1109)	4130	27		21.0 (966)	4649	21
30-31	50.5 (3372)	2856	118		40.3 (2722)	6427	42		28.2 (2074)	6415	32		22.8 (1587)	6416	25		17.5 (1232)	7144	17
32	43.5 (2298)	2446	94		36.6 (2324)	5289	44		26.2 (1665)	5279	32		20.9 (1157)	5280	22		18.3 (875)	5859	15
33	41.3 (3020)	3803	79		34.0 (2710)	8035	34		23.3 (1732)	8022	22		19.4 (1382)	8025	17		17.7 (1196)	9003	13
34	37.7 (4987)	6994	71		30.6 (4313)	14 486	30		20.7 (2726)	14 466	19		17.9 (2430)	14 471	17		14.6 (1866)	16 173	12
35	33.5 (6777)	11 362	60		27.7 (5883)	23 186	25		19.6 (3916)	23 147	17		16.3 (3030)	23 156	13		13.9 (2795)	25 842	11
36	32.3 (12 924)	22 946	56		25.8 (11 818)	46 543	25		18.6 (7925)	46 543	17		15.7 (6237)	46 543	13		12.7 (5338)	52 019	10
37	27.6 (24 897)	53 388	47		23.6 (22 954)	107 598	21		17.0 (16 197)	107 598	15		14.4 (12 938)	107 598	12		12.3 (11 727)	120 192	10
38	23.3 (50 096)	136 619	37		21.6 (51 351)	273 785	19		15.6 (36 194)	273 785	13		13.2 (29 287)	273 785	11		11.1 (26 035)	306 639	9
39	20.4 (70 567)	229 432	31		20.1 (75 329)	462 697	16		14.7 (55 379)	459 959	12		12.3 (44 628)	462 697	10		10.4 (39 538)	514 716	8
40	18.9 (79 680)	284 736	28		19.4 (89 689)	574 949	16		14.2 (63 762)	574 949	11		12.1 (52 498)	574 949	9		10.2 (46 033)	640 657	7
41	17.9 (53 818)	206 982	26		19.3 (63 940)	416 153	15		14.3 (47 575)	416 153	11		12.1 (38 250)	416 153	9		10.2 (34 461)	465 435	7
42	17.6 (10 717)	41 615	26		19.4 (12 757)	83 778	15		14.3 (9601)	83 778	12		12.0 (7477)	83 778	9		10.2 (6715)	93 361	7
Overall	35.1 (329 022)	1 006 135	33		30.6 (351 411)	2 030 477	17		21.5 (251 970)	2 027 619	12		17.5 (203 184)	2 030 372	10		14.6 (179 751)	2 265 473	8

% ≥1=percentage of children with one or more readmissions during age period; R=number of hospital readmissions; PY=total number of person years of follow-up (note that children are not at risk of subsequent hospital admission until they are discharged from hospital after birth admission); rate=rate per 100 person years.

In the unadjusted model, admission rates during childhood were inversely associated with gestational age (table 4). The admission rate of children born at less than 28 weeks’ gestational age had an admission rate five times higher than children born at 40 weeks (rate ratio 5.24, 95% confidence interval 4.91 to 5.60; P<0.001). The rate ratios decreased steadily by week of gestational age at birth. Even those born at 38 weeks (1.22, 1.19 to 1.24) and those born at 39 weeks (1.07. 1.05 to 1.09; P<0.001) had a significantly higher admission rate during childhood. Once the model was adjusted for other covariates (n=893 662), the rate ratios were slightly attenuated but remained statistically significant. Those born at less than 28 weeks had a rate ratio of 4.92 (95% confidence interval 4.58 to 5.30) compared with those born at 40 weeks. Those born at 38 weeks still had a rate almost 20% higher than those born at 40 weeks (1.19, 1.16 to 1.22; P<0.001). A total of 86 418 children were defined as high risk. When these children were excluded from the model, the adjusted RRs (n=814 852) decreased from 4.92 (4.58 to 5.30) to 3.24 (2.98 to 3.53) for those born at <28 weeks. The decline in rates was greater in those born very preterm, with only small differences observed in those born at early term (table 4).

**Table 4:** Unadjusted and adjusted rate ratios and 95% confidence intervals for hospital admissions during childhood by gestational age

Gestational age (weeks)	Unadjusted (n=1 018 136)	Adjusted* (n=893 662)	Adjusted excluding high risk children† (n=814 852)
<28	5.24 (4.91 to 5.60)	4.92 (4.58 to 5.30)	3.24 (2.98 to 3.53)
28-29	3.63 (3.33 to 3.97)	3.27 (2.99 to 3.57)	2.83 (2.66 to 3.01)
30-31	2.97 (2.53 to 3.49)	2.65 (2.22 to 3.17)	2.19 (2.09 to 2.31)
32	2.74 (2.42 to 3.10)	2.46 (2.17 to 2.78)	2.00 (1.89 to 2.11)
33	2.18 (2.05 to 2.31)	1.95 (1.83 to 2.07)	1.81 (1.72 to 1.90)
34	1.95 (1.84 to 2.06)	1.81 (1.71 to 1.92)	1.57 (1.52 to 1.63)
35	1.68 (1.61 to 1.75)	1.57 (1.50 to 1.64)	1.48 (1.43 to 1.53)
36	1.65 (1.58 to 1.73)	1.58 (1.51 to 1.65)	1.38 (1.35 to 1.42)
37	1.43 (1.39 to 1.47)	1.39 (1.35 to 1.43)	1.30 (1.28 to 1.33)
38	1.22 (1.19 to 1.24)	1.19 (1.16 to 1.22)	1.13 (1.12 to 1.15)
39	1.07 (1.05 to 1.09)	1.06 (1.04 to 1.08)	1.05 (1.04 to 1.06)
40	1.00	1.00	1.00
41	0.99 (0.97 to 1.01)	0.98 (0.96 to 1.01)	0.98 (0.97 to 0.99)
42	0.98 (0.95 to 1.01)	0.97 (0.94 to 1.00)	0.96 (0.94 to 0.98)

* Adjusted for maternal age at delivery, mother's country of birth, marital status, sex, small for gestational age, parity, delivery method, index of multiple deprivation score, child's ethnicity, and season of birth.

† High risk children defined as children with diagnosis of malignant neoplasm, blood disorder, cystic fibrosis, immune dysfunction, or congenital anomaly.

The population attributable fractions were highest during infancy and then declined over time. About 9.2%, 8.8%, and 4.2% of admissions during infancy were attributable to being born at 37, 38, and 39 weeks’ gestation, respectively (table 5); which equates to roughly 7373 excess admissions in infants each year.

**Table 5:** Population attributable fractions with 95% confidence intervals for each gestational age category and age at admission* (%)

Gestational age (weeks)	Age at admission					
	Overall	<1 year	1-2 years	3-4 years	5-6 years	7-10 years
<28	2.39 (2.35 to 2.44)	3.20 (3.14 to 3.24)	2.87 (2.81 to 2.92)	2.06 (1.98 to 2.13)	1.56 (1.47 to 1.64)	1.44 (1.34 to 1.53)
28-29	1.77 (1.69 to 1.83)	2.53 (2.47 to 2.59)	1.82 (1.75 to 1.89)	1.48 (1.39 to 1.56)	1.30 (1.20 to 1.39)	1.30 (1.18 to 1.39)
30-31	2.00 (1.76 to 2.20)	2.86 (2.77 to 2.93)	1.72 (1.62 to 1.81)	1.95 (1.83 to 2.05)	1.77 (1.65 to 1.88)	1.43 (1.29 to 1.56)
32	1.45 (1.32 to 1.57)	1.82 (1.74 to 1.89)	1.51 (1.42 to 1.59)	1.51 (1.40 to 1.61)	1.20 (1.09 to 1.30)	0.87 (0.73 to 0.99)
33	1.43 (1.33 to 1.52)	2.13 (2.03 to 2.22)	1.41 (1.29 to 1.51)	1.14 (1.01 to 1.27)	1.08 (0.93 to 1.21)	1.03 (0.86 to 1.18)
34	2.10 (1.95 to 2.25)	3.33 (3.20 to 3.44)	1.97 (1.83 to 2.11)	1.50 (1.32 to 1.67)	1.89 (1.71 to 2.06)	1.28 (1.05 to 1.48)
35	2.29 (2.12 to 2.46)	3.88 (3.72 to 4.03)	2.09 (1.91 to 2.27)	1.67 (1.44 to 1.89)	1.42 (1.17 to 1.65)	1.71 (1.44 to1.97)
36	4.30 (3.95 to 4.63)	6.71 (6.49 to 6.91)	4.12 (3.87 to 4.36)	3.42 (3.12 to 3.72)	3.03 (2.70 to 3.35)	2.79 (2.41 to 3.14)
37	5.93 (5.48 to 6.38)	9.19 (8.88 to 9.49)	4.98 (4.62 to 5.33)	4.69 (4.25 to 5.11)	4.37 (3.90 to 4.83)	5.03 (4.51 to 5.53)
38	5.87 (5.19 to 6.54)	8.76 (8.29 to 9.23)	5.13 (4.60 to 5.65)	4.68 (4.03 to 5.31)	4.18 (3.48 to 4.86)	4.99 (4.22 to 5.74)
39	2.69 (1.87 to 3.50)	4.19 (3.59 to 4.77)	1.51 (0.87 to 2.15)	2.96 (2.19 to 3.71)	2.37 (1.54 to 3.18)	2.46 (1.53 to 3.37)

* Population attributable fractions not calculated for 41 and 42 weeks as rate ratios are less than 1.

**Fig 2:**
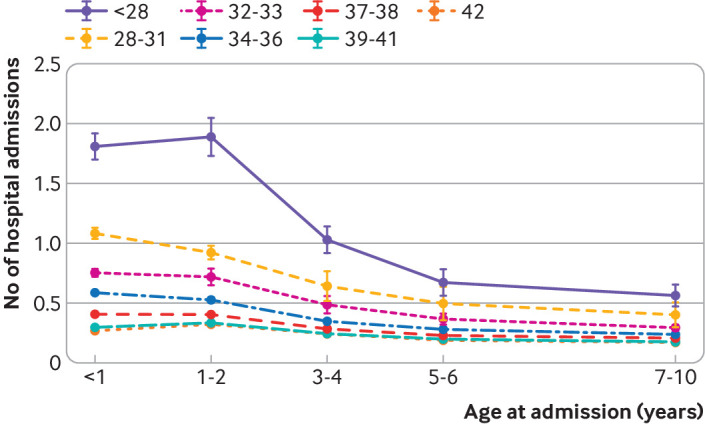
Mean hospital admissions by gestational age over time, adjusted for maternal age at delivery, mother's country of birth, marital status, sex, small for gestational age, parity, delivery method, index of multiple deprivation score, child's ethnicity, and month of birth

Evidence suggested a strong interaction between gestational age at birth and age at admission (P<0.001), with rate ratios for gestational age inversely associated with chronological age, particularly after the age of 2 (fig 2). During infancy, the admission rate was six times higher in babies born at less than 28 weeks than for those born at 40 weeks (rate ratio 6.34, 95% confidence interval 5.80 to 6.85) and it was 10% higher for those born at 39 weeks compared with 40 weeks (1.10, 1.08 to 1.11). However, by 7-10 years of age, the rate ratios had decreased to 3.28 (2.82 to 3.82) for those born at less than 28 weeks and 1.06 (1.03 to 1.08) for those born at 39 weeks. Before the age of 3, being born at 41 or 42 weeks’ gestation was associated with lower admission rates than for those born at 40 weeks. By 3-4 years of age, however, the rate ratios were close to one and no longer statistically significant (table 6). The results remained relatively stable in the other five sensitivity analyses (supplementary table S5) and when restricted to emergency hospital admissions (results not presented).

**Table 6:** Adjusted rate ratios* and 95% confidence intervals for hospital admissions during childhood, stratified by age at admission

Gestational age (weeks)	Age at admission				
	<1 year (n=893 662)	1-2 years (n=892 611)	3-4 years (n=892 112)	5-6 years (n=891 885)	7-10 years (n=891 745)
<28	6.34 (5.80 to 6.85)	5.78 (5.25 to 6.37)	4.35 (3.86 to 4.91)	3.49 (3.06 to 3.98)	3.28 (2.82 to 3.82)
28-29	4.26 (3.94 to 4.61)	3.32 (3.03 to 3.64)	2.91 (2.6 to 3.25)	2.69 (2.39 to 3.04)	2.70 (2.36 to 3.11)
30-31	3.37 (3.14 to 3.60)	2.41 (2.23 to 2.60)	2.61 (2.38 to 2.87)	2.52 (2.28 to 2.78)	2.21 (1.97 to 2.48)
32	2.84 (2.64 to 3.06)	2.48 (2.29 to 2.70)	2.47 (2.23 to 2.73)	2.25 (2.02 to 2.52)	1.87 (1.64 to 2.13)
33	2.40 (2.25 to 2.56)	1.92 (1.79 to 2.06)	1.76 (1.62 to 1.92)	1.72 (1.57 to 1.89)	1.69 (1.52 to 1.88)
34	2.30 (2.19 to 2.41)	1.75 (1.66 to 1.85)	1.58 (1.48 to 1.69)	1.75 (1.63 to 1.88)	1.49 (1.37 to 1.61)
35	1.98 (1.90 to 2.06)	1.51 (1.45 to 1.58)	1.41 (1.33 to 1.49)	1.35 (1.27 to 1.43)	1.43 (1.33 to 1.52)
36	1.92 (1.87 to 1.98)	1.55 (1.50 to 1.60)	1.45 (1.39 to 1.51)	1.40 (1.34 to 1.46)	1.37 (1.30 to 1.43)
37	1.63 (1.60 to 1.66)	1.32 (1.29 to 1.35)	1.30 (1.27 to 1.34)	1.28 (1.25 to 1.32)	1.33 (1.29 to 1.37)
38	1.29 (1.27 to 1.31)	1.16 (1.14 to 1.18)	1.15 (1.13 to 1.17)	1.13 (1.11 to 1.16)	1.16 (1.13 to 1.19)
39	1.10 (1.08 to 1.11)	1.03 (1.02 to 1.05)	1.07 (1.05 to 1.09)	1.05 (1.03 to 1.07)	1.06 (1.03 to 1.08)
40	1.00	1.00	1.00	1.00	1.00
41	0.92 (0.91 to 0.93)	0.97 (0.96 to 0.99)	1.03 (1.01 to 1.05)	1.01 (0.99 to 1.03)	1.03 (1.01 to 1.05)
42	0.92 (0.89 to 0.94)	0.96 (0.93 to 0.99)	1.02 (0.98 to 1.05)	0.97 (0.94 to 1.00)	1.00 (0.96 to 1.04)

* Adjusted for mother’s age at delivery, mother's country of birth, marital status, sex, small for gestational age, parity, delivery method, index of multiple deprivation score, child's ethnicity, and month of birth.

**Fig 3:**
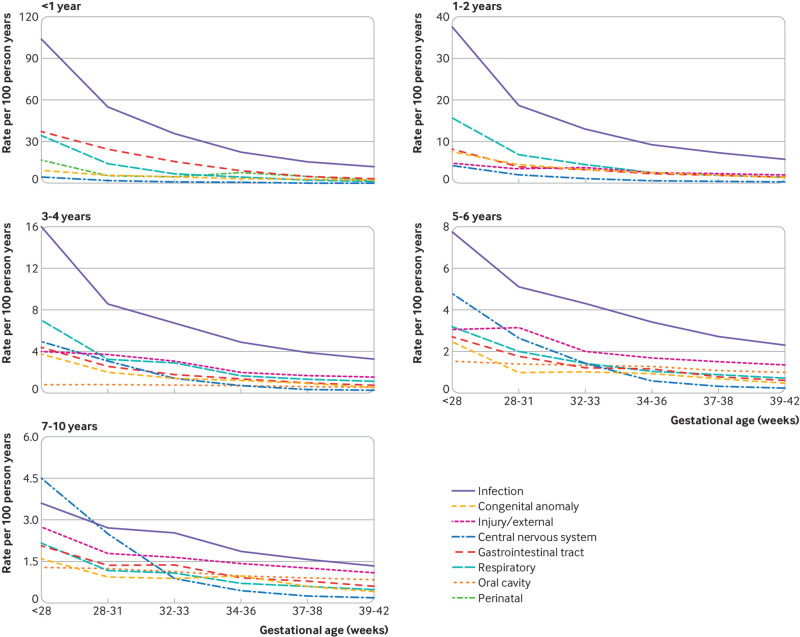
Crude hospital admission rates per 100 person years by cause of morbidity, gestational age, and age at admission

Figure 3 and supplementary table S7 present crude hospital admission rates per 100 person years for key causes of morbidity by gestational age (<28, 28-31, 32-33, 34-36, 37-38, and 39-42 weeks) and age at admission (<1, 1-2, 3-4, 5-6, and 7-10). The highest hospital admission rates were seen in infancy, and these rates continued to decline with increasing age.

At all ages, infection was the most common cause of admission and the rate increased markedly as gestational age decreased, suggesting that the excess admissions in children born before 39-42 weeks were largely due to infection—in particular, respiratory infections (fig 3). In infancy, excess admissions were also seen in children born before 39-42 weeks due to non-infection gastrointestinal tract conditions in children born at less than 37 weeks’ gestation and non-infection respiratory causes in children born at less than 34 weeks. At 1-2 and 3-4 years, excess admissions due to non-infection respiratory causes were seen in children born at less than 34 weeks’ gestation. At ages 5-6 and 7-10, injuries were the second most common cause of admission (after infection) and accounted for a large proportion of excess admissions in children born before 39-42 weeks, particularly in children born at less than 32 weeks. In children born at less than 34 weeks, however, central nervous system causes also accounted for many excess admissions and the most common ICD-10 codes were related to epilepsy and cerebral palsy. Crude rates are presented in supplementary table S7.

Almost all (97%) infection related admissions during infancy were emergencies, but this declined over time to 63% by 7-10 years of age. Similar patterns were observed for admissions for non-infection respiratory disease and non-infection gastrointestinal tract disease.

## Discussion

### Principal findings

The results from our study indicate that gestational age at birth is a strong predictor of severe morbidity throughout childhood in England. Children had a consistently lower admission rate with each additional week of gestational age at birth. Adjusting for other prognostic characteristics altered the strength of this association little, as did the various sensitivity analyses conducted. Importantly, the association between gestational age and severe morbidity ameliorated over time, with the sharpest decline in rates seen after age 2, particularly in those born extremely preterm. However, in the age stratified analysis, the effect of gestational age persisted in later stages of childhood, even for those born at 38 and 39 weeks.

The results were attenuated when high risk children were excluded, with the most marked decrease seen in those born at 32 weeks of gestation or earlier, suggesting there is heterogeneity within gestational age groups. Infection accounted for most hospital admissions at all ages and there was a strong association between infection related admissions and age at admission. Other common causes of admission were respiratory (non-infection) and gastrointestinal tract (non-infection) related, especially in younger children, and injuries and central nervous system related in older children. Many of these cause specific rates showed a marked increase as gestational age at birth decreased. Among those born extremely preterm, the most common cause of admission by age 7-10 was related to problems of the central nervous system, primarily cerebral palsy and epilepsy.

### Strengths and limitations

In this large study we investigated the association between gestational age at birth and longer term health outcomes in England. A key strength of this study is its large size, providing sufficient power to investigate the effects across the full spectrum of gestational age, thereby detecting even small differences in hospital admission rates between gestational age categories. Additionally, we analysed up to 10 years of follow-up data, enabling us to estimate hospital admission trajectories through early and mid-childhood. Finally, the use of routinely collected data means that the results are largely unaffected by recall and social desirability bias.^31^

The study has a number of limitations. HES data are collected primarily for financial reimbursement rather than for research, leading to large variations in the quality and completeness of particular data fields.^15^ Linkage to birth registration data made it possible to overcome some of these concerns about quality, but for some fields, such as parity and labour induction, it was not possible to recover or validate some of these missing or poor quality data. We conducted two sensitivity analyses, however, where we adjusted for labour induction and excluded hospitals that were defined as poor reporters of parity, but neither of these approaches had much effect on the results. The HES dataset also does not provide information on migration from the NHS, therefore, it is possible that children could have moved out of England or transferred care to private or military hospitals, which would not be reflected in the admission rates. This migration would comprise a small proportion of the sample population, however, and is unlikely to change the estimates generated. In addition, it was not possible to adjust for particular confounders and mediators demonstrated in other research studies as predictive of adverse long term sequelae in children, such as maternal smoking, breastfeeding, individual level markers of social deprivation, or maternal conditions associated with preterm birth, as these variables are not recorded reliably or at all in birth registration records or in HES. Moreover, the cause of admission was defined using the primary diagnosis code. This code is often the main reason for admission, but in some cases it will reflect the most expensive diagnosis rather than the key reason for admission. Finally, a complete case analysis was conducted rather than using multiple imputation to deal with the missing data. The estimates remained fairly stable in all models, but we cannot rule out the potential for bias in the results from the fully adjusted model due to excluding participants with missing data.

### Comparison with other studies

Many studies have investigated the effect of preterm birth on outcomes for offspring, but few studies have examined all cause, long term hospital admissions across the whole spectrum of gestational age, particularly in UK populations. Although UK studies all report a decline in hospital admission rates with increasing gestational age, they either group gestational age in broad categories^4^ ^12^ ^32^ or focus on specific causes of admission to hospital.^9^ ^13^ ^32^

Our study found that 52% of children had at least one hospital admission by 10 years of age. Two Australian studies reported that 62%^5^ and 56%^14^ of study participants had at least one hospital admission by 18 years of age, with the vast majority of admissions occurring before age 12. In our study, very and extremely preterm babies had much higher admission rates during infancy than those born full term, which is consistent with published data. Similar studies in Australia^14^ and France^33^ have reported admission rates in extremely and very preterm babies to be seven and three times higher than in those born full term, respectively. Other studies conducted in Australia^34^ ^35^ and the US^6^ found very preterm babies were two to three times more likely than those born at full term to be admitted to hospital during infancy. These results are important contributors to this subject area, but differences in health and social care systems mean results might not be generalisable to UK populations.

Few UK based studies have explored how risk of admission changes during childhood. One UK study^12^ found that compared with full term babies, very preterm infants were more than 13 times more likely by 9 months of age, and six time more likely at age 5, to have three or more hospital admissions.^12^ A study conducted in Australia^14^ reported lower admission rates among older children; compared with full term infants, admission rates declined from 7.77 in infancy to 2.94 at 5-12 years of age. Similar findings have been reported in other studes.^5^ Our findings, with narrower age groups, suggest that the sharpest decline in hospital admissions is seen after the age of 2, particularly in those born extremely preterm.

The increased risk of admission in children born at 38 and 39 gestational weeks is consistent with other research. Globally, the rates of births before 40 weeks’ gestation have been increasing over the past 20 years, and the average gestational age at delivery has consequently decreased from 40 to 39 weeks.^36^ ^37^ This decline is attributed to an increase in caesarean sections and induction rates, and a clinical perception that there is little risk from being born a week or two before 40 weeks’ gestation.^38^ Although the rates of admission are only slightly higher for those born at 38 and 39 weeks than for those born at 40 weeks, they account for 37% of births within our cohort. About 13% of admissions (population attributable fractions 8.76+4.19) during infancy could be avoided if these babies were born at 40 weeks’ gestation. This early induction must also be balanced with the risk of stillbirth, and short term risks and safety of the mother and baby. Medically indicated birth before 40 weeks’ gestation will be due to clinical concern about the health of either the mother or baby. Therefore, it is possible that poorer outcomes in these children are as much, or more, related to the effects of maternal illness or complications of pregnancy as to early term birth itself.^39^

Admission rates in children born post-term (≥42 weeks’ gestation) were slightly lower than those for full term infants, but in stratified analyses, this pattern disappeared after age 2. Similar findings were also seen in a number of other studies,^7^ ^34^ ^35^ although it was not possible to see how this effect changed over time. In contrast, a study conducted in Australia^14^ reported an increased rate of admission to hospital in children born at 42 weeks or later when stratified by age, with the highest rates of admission seen within the first year of life. Our results suggest that not all post-term children will have poorer outcomes than those born at full term, and future research should explore the effect of post-term birth by each week of gestational age.

Our study showed that infections, particularly respiratory infections, account for most hospital admissions during childhood. Infection related admissions were strongly associated with gestational age, and although rates declined with age, they were still the most common cause of admission at 7-10 years. Preterm infants are at increased risk of infection owing to their immature immune systems, which continue to develop at a slower rate throughout childhood in comparison with their full term peers.^40^ These infants are also at increased risk of impaired lung function, leaving them particularly vulnerable to respiratory problems.^41^ Our results suggest that even children born early term are at increased risk of infections throughout early childhood, particularly during infancy, in comparison with those born at full term.

Our findings suggest that children born at less than 34 weeks’ gestation are particularly at risk of central nervous system related admissions, specifically epilepsy and cerebral palsy, and in extremely preterm children, these diseases were the most common causes of hospital admission at age 7-10. A study conducted in Australia reported similar findings^5^ and an increased risk of brain injury, such as intraventricular haemorrhage and periventricular leukomalacia, or severe illness in the early neonatal period, might be responsible for this association in children born at less than 34 weeks.^42^

It is also striking that other causes of admission showed a strong association with gestational age. For example, rates of injury related admissions, which were relatively common from age 3-10, increased as gestational age decreased. This increase has been noted in a similar study,^5^ and it is possible that neurobehavioural disorders and impaired cognitive function associated with preterm birth might account for this.^43^

Although preterm children often have physiological characteristics which cause morbidity, it is also possible that the knowledge that a child was born preterm had a role in their admission to hospital. Vulnerable child syndrome describes children who are perceived, usually by parents, to be at greater risk of developmental delays and adverse health outcomes, and preterm birth is a known risk factor.^44^ Children with increased perceived vulnerability are likely to have high numbers of emergency department visits during childhood,^44^ and a study conducted in Australia found that clinicians often overestimated the adverse outcomes of extremely preterm infants.^45^ It is plausible, therefore, that the perception of vulnerability could be driving some of the excess admissions in children who are preterm. 

### Implications

Overall, the findings from this study have illustrated the need for strategies aimed at the prevention and management of infections in children born preterm and close to term. In addition, it is particularly important for children born at less than 34 weeks, who continue to have increased admissions due to central nervous system causes up to age 10, to be monitored closely. The National Institute for Health and Care Excellence recommends that children born before 30 weeks’ gestation, and those born between 30 and 36 weeks’ gestation who have additional risk factors, should be monitored and assessed up to age 2 for developmental deficits, and up to age 4 for those at highest risk (born <28 weeks’ gestation). Risk factors in more mature preterm and early term births have not been clearly elucidated, and a report found that there was wide variation in the provision of clinical surveillance across the UK.^46^ Many medically indicated births before 40 weeks cannot be avoided as risks of delaying delivery often outweigh potential benefits. If the clinical decision for early delivery is not clear cut—for example, whether low risk women aged 35 and over should be induced before 40 weeks’ gestation,^47^ ^48^ the long term risks should be discussed with parents. Neonatal, paediatric, and primary care clinicians should be aware of the increased likelihood of infections, respiratory and gastrointestinal tract problems in children born even a small number of weeks early, in order to advise parents appropriately.

The findings indicate a need for research to examine the full spectrum of gestational age, week by week, with a focus on understanding the long term health outcomes of early term, late term, and post-term births. A small group of children born very and extremely preterm had no hospital admissions up to the age of 10, which suggests that children might have different risk profiles within gestational age categories. Exploration of this heterogeneity might make it possible to identify factors associated with better long term outcomes within these groups. In addition, the small, but increased rates of hospital admission in children born a week or two early suggest there should be a move away from the assumption that birth at 39 weeks carries no additional risk to the child. Finally, research is needed to understand more fully clinical decision making for hospital admissions among children born before 40 weeks’ gestation, as these decisions could be amenable to intervention and therefore important in reducing admission rates.

### Conclusions

The findings from this study indicate that gestational age at birth is a strong predictor of childhood morbidity, with those born extremely preterm being at the greatest risk of hospital admission throughout childhood. The risk of hospital admission associated with gestational age decreased over time, particularly after age 2; however, an excess risk remained up to age 10, even for children born at 38 and 39 weeks’ gestation. Although the excess risk at 38 and 39 weeks was relatively small, the large number of babies born globally at these gestational ages suggests that they are likely to be a considerable clinical and economic burden. Strategies aimed at the prevention and management of childhood infections should target preterm children and those born close to full term. Future research should consider gestational age as a continuum and explore it for outcomes week by week.

**What is already known on this topic**

Preterm birth is a major contributor to childhood morbidity
Few studies have investigated the long term health consequences of the full spectrum of gestational age at birth in large, population based studies in the UK
Existing evidence suggests that the risk of morbidity associated with preterm birth declines as children grow up, but it remains unclear at what age this begins to happen and how these changes vary by week of gestational age at birth

**What this study adds**

Using a large, population based, record linkage dataset, the findings from our study indicate that gestational age at birth is inversely associated with hospital admissions throughout childhood and that the effect of gestational age decreases over time, with the highest rates within the first two years after birth for all gestational ages
Although the excess risk of admission to hospital in those born at 37, 38, and 39 weeks was relatively small, 42% of children were born at these gestational ages in our cohort, representing many potentially vulnerable children
Hospital admissions due to infections were strongly associated with gestational age and were the main driver of excess hospital admissions at all ages, but particularly so during infancy

## Web extra

Extra material supplied by authors

Web appendix 1: ICD10 codes: categories of admission

Web appendix: Supplementary information

## Notes

Contributors: MAQ (guarantor) designed the study with input from EB, CC, SJ, JJK, AJM, SP, and ORA. MAQ and AJM were responsible for the acquisition of the data. VC (guarantor), MAQ and AJM were involved in data cleaning with input from Rod Gibson (data management consultant) and Nirupa Dattani (data analyst at City University). Statistical analysis was performed by VC and MAQ. VC, EB, CC, SJ, JJK, AM, SP, OR-A, and MAQ were all involved in the interpretation of the data. VC was responsible for the initial draft of the manuscript. EB, CC, SJ, JJK, AM, SP, OR-A, and MAQ reviewed and contributed to drafts of the manuscript, and all authors reviewed the final version. The corresponding author attests that all listed authors meet authorship criteria and that no others meeting the criteria have been omitted.

Ethical approval: Ethical approval for this study was granted by the Health Research Authority research ethics committee (South West – Frenchay; REC reference 15/SW/0294) and was provided by the Health Research Authority following Advice from the Confidentiality Advisory Group (CAG reference 15/CAG/0196). The TIGAR study used data linked as part of a previous study led by City, University of London. For that study permission to use patient identifiable data without consent under regulation 5 of the Health Service (control of patient information) Regulations 2002 (“section 251 support”) was initially granted by the Patient Information Advisory Group PIAG 2-10(g)/2005. Renewals and amendments and a second permission, CAG 9-08(b)2014, under regulation 5 of the Health Service (control of patient information) Regulations 2002 (or “same legislation”) were granted by the Secretary of State for Health and the Health Research Authority following advice from the CAG. A second permission CAG 9-08(b)2014 to use patient identifiable data without consent under regulation 5 of the Health Service (control of patient information) Regulations 2002 and create a research database held at the ONS for analyses relating to inequalities in the outcome of pregnancy and to inform maternity service users about the outcome of midwifery, obstetric, and neonatal care was granted by the Health Research Authority. For the TIGAR study, permission to use patient identifiable data without consent under regulation 5 of the Health Service (control of patient information) Regulations 2002 was granted by the Secretary of State for Health and the Health Research Authority following advice from the CAG (CAG reference 15/CAG/0196). Permission from the Health and Social Care Information Centre for preliminary quality assurance work was included in data sharing agreement NIC-273840-N0N0 N. Permission for the subsequent analyses in the TIGAR study was included in data sharing agreement NIC-09637-Y8T1N-v1.3.

Data sharing: The authors do not have permission to supply data or identifiable information to third parties, including other researchers, but the team at City, University of London has permission under regulation 5 of the Health Service (control of patient information) Regulations 2002 to analyse patient identifiable data for England and Wales without consent and create a research database that could be accessed by other researchers using the SRS at the ONS. The TIGAR team has permission under regulation 5 of the Health Service (control of patient information) Regulations 2002 to analyse these data. Anyone wishing to access the linked datasets for research purposes should apply via the CAG to the Health Research Authority to access patient identifiable data without consent and then to the ONS and NHS Digital. In the first instance, enquiries about access to the data should be addressed to Alison Macfarlane.

The lead author (the manuscript’s guarantor) affirms that the manuscript is an honest, accurate, and transparent account of the study being reported; that no important aspects of the study have been omitted; and that any discrepancies from the study as planned (and, if relevant, registered) have been explained.

Dissemination to participants and related patient and public communities: As study data were pseudonymised, it is not possible to send findings directly to the study participants. The findings from the TIGAR study (including those from this paper) will be disseminated via our PPI parent group and the charities which have supported the study. The results will also be made available on the TIGAR webpage https://www.npeu.ox.ac.uk/tigar.

Provenance and peer review: Not commissioned; externally peer reviewed.
