# Supplementary material for: Gestational age and hospital admissions during childhood: population based, record linkage study in England (TIGAR study)
Source: BMJ. 2020 Nov 25;371:m4075. doi: 10.1136/bmj.m4075 (PMC7687266; doi:10.1136/bmj.m4075)
Supplement: Supplementary file 1 — Web appendix 1: ICD10 codes: categories of admission [file coav055130.ww1.pdf]

ICD10 codes: categories of admission

Infection

A02.1 Salmonella sepsis
A17.0 Tuberculous meningitis
A17.1 Meningeal tuberculoma
A17.8 Other tuberculosis of nervous system
A17.9 Tuberculosis of nervous system, unspecified
A18.0 Tuberculosis of bones and joints
A19.0 Acute miliary tuberculosis of a single specified site
A19.1 Acute miliary tuberculosis of multiple sites
A20.3 Plague meningitis
A20.7 Septicemic plague
A21.7 Generalized tularaemia
A22.7 Anthrax sepsis
A23.0 Brucellosis due to Brucella melitensis
A23.1 Brucellosis due to Brucella abortus
A23.2 Brucellosis due to Brucella suis
A23.3 Brucellosis due to Brucella canis
A23.8 Other brucellosis
A23.9 Brucellosis, unspecified
A24.1 Acute and fulminating melioidosis
A25.0 Spirillosis
A25.1 Streptobacillosis
A25.9 Rat-bite fever, unspecified
A32.1 Listerial meningitis and meningoencephalitis
A32.7 Listerial sepsis
A39.0 Meningococcal meningitis
A39.1 Waterhouse-Friderichsen syndrome
A39.2 Acute meningococcaemia
A39.3 Chronic meningococcaemia
A39.4 Meningococcemia, unspecified
A39.5 Meningococcal heart disease
A39.8 Other meningococcal infections
A39.8Other meningococcal infections
A39.9 Meningococcal infection, unspecified
A40.0 Sepsis due to streptococcus, group A
A40.1 Sepsis due to streptococcus, group B
A40.2 Sepsis due to streptococcus, group D
A40.3 Sepsis due to Streptococcus pneumoniae
A40.8 Other streptococcal sepsis
A40.9 Streptococcal sepsis, unspecified
A41.0 Sepsis due to Staphylococcus aureus
A41.1 Sepsis due to other specified staphylococcus
A41.2 Sepsis due to unspecified staphylococcus
A41.3 Sepsis due to Hemophilus influenzae
A41.4 Sepsis due to anaerobes
A41.51 Sepsis due to Escherichia coli [E. Coli]
A41.52 Sepsis due to Pseudomonas
A41.58 Sepsis due to other Gram-negative organisms
A41.8 Other specified septicaemia
A41.9 Sepsis, unspecified
A44.0 Systemic bartonellosis
A48.3 Toxic shock syndrome

A52.0 Cardiovascular syphilis
A52.1 Symptomatic neurosyphilis
A52.1 (no description found)
A52.2 Asymptomatic neurosyphilis
A52.3 Neurosyphilis, unspecified
A52.7 Other symptomatic late syphilis
A52.7 (no description found)
A52.8 Late syphilis, latent
A65 Nonvenereal syphilis
A74.0 Chlamydial conjunctivitis
A74.8 Other chlamydial diseases
A78 Q fever
A79.0 Trench fever
A79.1 Rickettsialpox due to Rickettsia akari
A79.8 Other specified rickettsioses
A79.9 Rickettsiosis, unspecified
B95.1 Streptococcus, group B, as the cause of diseases classified elsewhere
G00.0 Haemophilus meningitis
G00.1 Pneumococcal meningitis
G00.1 (no description found)
G00.2 Streptococcal meningitis
G00.3 Staphylococcal meningitis
G00.8 Other bacterial meningitis
G00.9 Bacterial meningitis, unspecified
G01 Meningitis in bacterial diseases classified elsewhere
G03.1 Chronic meningitis
G05.0 Encephalitis, myelitis and encephalomyelitis in bacterial diseases classified elsewhere
G06.0 Intracranial abscess and granuloma
G06.1 Intraspinal abscess and granuloma
G06.2 Extradural and subdural abscess, unspecified
G07 Intracranial and intraspinal abscess and granuloma in disease classified elsewhere
I30.1 Infective pericarditis
I32.0 Pericarditis in bacterial diseases classified elsewhere
I33.0 Acute and subacute infective endocarditis
I41.0 Myocarditis in bacterial diseases classified elsewhere
J39.0 Retropharyngeal and parapharyngeal abscess
J39.1 Other abscess of pharynx
J85.3 Abscess of mediastinum
M00.90 Pyogenic arthritis, unspecified, multiple sites
M00.91 Pyogenic arthritis, unspecified, shoulder region
M00.92 Pyogenic arthritis, unspecified, upper arm
M00.93 Pyogenic arthritis, unspecified, forearm
M00.94 Pyogenic arthritis, unspecified, hand
M00.95 Pyogenic arthritis, unspecified, pelvic region and thigh
M00.96 Pyogenic arthritis, unspecified, lower leg
M00.97 Pyogenic arthritis, unspecified, ankle and foot
M00.98 Pyogenic arthritis, unspecified, other site
M00.99 Pyogenic arthritis, unspecified, site unspecified
M01.0 Meningococcal arthritis
M01.1 Tuberculous arthritis
M46.2 Osteomyelitis of vertebra
M46.3 Infection of intervertebral disc (pyogenic)
M46.4 Discitis, unspecified
M46.5 Other infective spondylopathies

M49.0 Tuberculosis of spine
M49.1 Brucella spondylitis
M49.2 Enterobacterial spondylitis
M49.3 Spondylopathy in other infectious and parasitic diseases classified elsewhere
M72.6 Necrotizing fasciitis
M73.0 Gonococcal bursitis
M73.1 Syphilitic bursitis
M86.0 Acute haematogenous osteomyelitis
M86.10 Other acute osteomyelitis, unspecified site
M86.11 Other acute osteomyelitis, shoulder region
M86.12 Other acute osteomyelitis, upper arm
M86.13 Other acute osteomyelitis, forearm
M86.14 Other acute osteomyelitis, hand
M86.15 Other acute osteomyelitis, pelvic region and thigh
M86.16 Other acute osteomyelitis, lower leg
M86.17 Other acute osteomyelitis, ankle and foot
M86.18 Other acute osteomyelitis, other site
M86.19 Other acute osteomyelitis, multiple sites
M86.2 Subacute osteomyelitis
M86.4 Chronic osteomyelitis with draining sinus
M86.5 Other chronic osteomyelitis
M86.60 Other chronic osteomyelitis, unspecified site
M86.67 Other chronic osteomyelitis, ankle and foot
M86.68 Other chronic osteomyelitis, other site
M86.69 Other chronic osteomyelitis, multiple sites
M86.8 Other osteomyelitis
M86.90 Unspecified osteomyelitis, multiple sites
M86.91 Unspecified osteomyelitis, shoulder region
M86.92 Unspecified osteomyelitis, upper arm
M86.93 Unspecified osteomyelitis, forearm
M86.94 Unspecified osteomyelitis, hand
M86.95 Unspecified osteomyelitis, pelvic region and thigh
M86.96 Unspecified osteomyelitis, lower leg
M86.97 Unspecified osteomyelitis, ankle and foot
M86.98 Unspecified osteomyelitis, other site
M86.99 Unspecified osteomyelitis, site unspecified
M90.0 Tuberculosis of bone
P36.0 Sepsis of newborn due to streptococcus, group B
P36.1 Sepsis of newborn due to other and unspecified streptococci
P36.2 Sepsis of newborn due to Staphylococcus aureus
P36.3 Sepsis of newborn due to other and unspecified staphylococci
P36.4 Sepsis of newborn due to Escherichia coli
P36.5 Sepsis of newborn due to anaerobes
P36.8 Other bacterial sepsis of newborn
P36.9 Bacterial sepsis of newborn, unspecified
P37.0 Congenital tuberculosis
P37.2 Neonatal (disseminated) listeriosis

A00.0 Cholera due to Vibrio cholerae 01, biovar cholerae
A00.1 Cholera due to Vibrio cholerae 01, biovar eltor
A00.9 Cholera, unspecified
A01.0 Typhoid fever
A01.1 Paratyphoid fever A
A01.2 Paratyphoid fever B

A01.3 Paratyphoid fever C
A01.4 Paratyphoid fever, unspecified
A02.0 Salmonella enteritis
A02.2 Localised salmonella infections
A02.8 Other specified salmonella infections
A02.9 Salmonella infection, unspecified
A03.0 Shigellosis due to Shigella dysenteriae
A03.1 Shigellosis due to Shigella flexneri
A03.2 Shigellosis due to Shigella boydii
A03.3 Shigellosis due to Shigella sonnei
A03.8 Other shigellosis
A03.9 Shigellosis, unspecified
A04.0 Enteropathogenic Escherichia coli infection
A04.1 Enterotoxigenic Escherichia coli infection
A04.2 Enteroinvasive Escherichia coli infection
A04.3 Enterohemorrhagic Escherichia coli infection
A04.4 Other intestinal Escherichia coli infections
A04.5 Campylobacter enteritis
A04.6 Enteritis due to Yersinia enterocolitica
A04.7 Enterocolitis due to Clostridium difficile
A04.8 Other specified bacterial intestinal infections
A04.9 Bacterial intestinal infection, unspecified
A05.0 Foodborne staphylococcal intoxication
A05.1 Botulism food poisoning
A05.2 Foodborne Clostridium perfringens [Clostridium welchii] intoxication
A05.3 Foodborne Vibrio parahaemolyticus intoxication
A05.4 Foodborne Bacillus cereus intoxication
A05.8 Other specified bacterial foodborne intoxications
A05.9 Bacterial foodborne intoxication, unspecified
A06.0 Acute amebic dysentery
A06.1 Chronic intestinal amebiasis
A06.2 Amebic nondysenteric colitis
A06.3 Amoeboma of intestine
A06.4 Amebic liver abscess
A06.9 Amebiasis, unspecified
A07.0 Balantidiasis
A07.1 Giardiasis [lambliasis]
A07.2 Cryptosporidiosis
A07.3 Isosporiasis
A07.8 Other specified protozoal intestinal diseases
A07.9 Protozoal intestinal disease, unspecified
A08.0 Rotaviral enteritis
A08.1 Acute gastroenteropathy due to Norwalk agent
A08.2 Adenoviral enteritis
A08.3 Other viral enteritis
A08.4 Viral intestinal infection, unspecified
A08.5 Other specified intestinal infections
A09 Infectious gastroenteritis and colitis, unspecified
A09.0 Other and unspecified gastroenteritis and colitis of infectious origin
A09.9 Gastroenteritis and colitis of unspecified origin
A21.3 Gastrointestinal tularemia
A22.2 Gastrointestinal anthrax
B37.88 Candidiasis of other sites
B71.0 Hymenolepiasis

B71.9 Cestode infection, unspecified
B76.0 Ancylostomiasis
B78.9 Strongyloidiasis, unspecified
B81.0 Anisakiasis
B96.81 Helicobacter pylori [H. pylori] as the cause of diseases classified to other chapters
K52.8 Other specified noninfective gastroenteritis and colitis

A06.5 Amebic lung abscess
A15.0 Tuberculosis of lung
A15.1 Tuberculosis of lung, confirmed by culture only
A15.2 Tuberculosis of lung, confirmed histologically
A15.3 Tuberculosis of lung, confirmed by unspecified means
A15.4 Tuberculosis of intrathoracic lymph nodes
A15.5 Tuberculosis of larynx, trachea and bronchus
A15.6 Tuberculosis pleurisy
A15.7 Primary respiratory tuberculosis
A15.8 Other respiratory tuberculosis
A15.9 Respiratory tuberculosis unspecified, confirmed bacteriologically and histologically
A16.0 Tuberculosis of lung, bacteriologically and histologically negative
A16.1 Tuberculosis of lung, bacteriological and histological examination not done
A16.2 Tuberculosis of lung, without mention of bacteriological or histological confirmation
A16.3 Tuberculosis of intrathoracic lymph nodes, without mention of bacteriological or histological confirmation
A16.4 Tuberculosis of larynx, trachea and bronchus, without mention of bacteriological or histological confirmation
A16.5 Tuberculous pleurisy, without mention of bacteriological or histological confirmation
A16.7 Primary respiratory tuberculosis, without mention of bacteriological or histological confirmation
A16.8 Other respiratory tuberculosis, without mention of bacteriological or histological confirmation
A16.9 Respiratory tuberculosis unspecified, without mention of bacteriological or histological confirmation
A19.2 Acute miliary tuberculosis, unspecified
A19.8 Other miliary tuberculosis
A19.9 Miliary tuberculosis, unspecified
A20.2 Pneumonic plague
A21.2 Pulmonary tularemia
A22.1 Pulmonary anthrax
A24.0 Glanders
A31.0 Pulmonary mycobacterial infection
A37.0 Whooping cough due to Bordetella pertussis
A37.1 Whooping cough due to Bordetella parapertussis
A37.8 Whooping cough due to other Bordetella species
A37.9 Whooping cough, unspecified
A42.0 Pulmonary actinomycosis
A48.1 Legionnaires disease
A70 Chlamydia psittaci infections
A70 (no description found)
B01.2 Varicella pneumonia
B05.2 Measles complicated by pneumonia
B34.0 Adenovirus infection, unspecified
B37.1 Pulmonary candidiasis
B38.0 Acute pulmonary coccidioidomycosis
B38.1 Chronic pulmonary coccidioidomycosis
B38.2 Pulmonary coccidioidomycosis, unspecified
B39.0 Acute pulmonary histoplasmosis capsulati
B39.1 Chronic pulmonary histoplasmosis capsulati
B39.2 Pulmonary histoplasmosis capsulati, unspecified

B39.3 Disseminated histoplasmosis capsulati
B39.4 Histoplasmosis capsulati, unspecified
B39.5 Histoplasmosis duboisii
B39.9 Histoplasmosis, unspecified
B40.0 Acute pulmonary blastomycosis
B40.1 Chronic pulmonary blastomycosis
B40.2 Pulmonary blastomycosis, unspecified
B40.3 Cutaneous blastomycosis
B40.7 Disseminated blastomycosis
B40.8 Other forms of blastomycosis
B41.0 Pulmonary paracoccidioidomycosis
B42.0 Pulmonary sporotrichosis
B44.0 Invasive pulmonary aspergillosis
B44.1 Other pulmonary aspergillosis
B45.0 Pulmonary cryptococcosis
B58.3 Pulmonary toxoplasmosis
B59 Pneumocystosis
J05.0 Acute obstructive laryngitis [croup]
J09 Influenza due to certain identified influenza virus
J10.0 Influenza with pneumonia, influenza virus identified
J10.1 Influenza due to other influenza virus with respiratory manifestations
J11.0 Influenza with pneumonia, virus not identified
J11.1 Influenza with other respiratory manifestations, virus not identified
J12.0 Adenoviral pneumonia
J12.1 Respiratory syncytial virus pneumonia
J12.2 Parainfluenza virus pneumonia
J12.3 Human metapneumovirus pneumonia
J12.8 Other viral pneumonia
J12.9 Viral pneumonia, unspecified
J13 Pneumonia due to Streptococcus pneumoniae
J14 Pneumonia due to Hemophilus influenzae
J15.0 Pneumonia due to Klebsiella pneumoniae
J15.1 Pneumonia due to Pseudomonas
J15.2 Pneumonia due to staphylococcus
J15.3 Pneumonia due to streptococcus, group B
J15.4 Pneumonia due to other streptococci
J15.5 Pneumonia due to Escherichia coli
J15.6 Pneumonia due to other aerobic Gram-negative bacteria
J15.7 Pneumonia due to Mycoplasma pneumoniae
J15.8 Pneumonia due to other specified bacteria
J15.9 Unspecified bacterial pneumonia
J16.0 Chlamydial pneumonia
J16.8 Pneumonia due to other specified infectious organisms
J17.0 Pneumonia in bacterial diseases classified elsewhere
J17.1 Pneumonia in viral diseases classified elsewhere
J17.2 Pneumonia in mycoses
J17.3 Pneumonia in parasitic diseases
J17.8 Pneumonia in other diseases classified elsewhere
J18.0 Bronchopneumonia, unspecified organism
J18.1 Lobar pneumonia, unspecified
J18.8 Other pneumonia, unspecified organism
J18.9 Pneumonia, unspecified
J20.0 Acute bronchitis due to Mycoplasma pneumoniae
J20.1 Acute bronchitis due to Hemophilus influenzae

J20.2 Acute bronchitis due to streptococcus
J20.3 Acute bronchitis due to coxsackievirus
J20.4 Acute bronchitis due to parainfluenza virus
J20.5 Acute bronchitis due to respiratory syncytial virus
J20.6 Acute bronchitis due to rhinovirus
J20.7 Acute bronchitis due to echovirus
J20.8 Acute bronchitis due to other specified organisms
J20.9 Acute bronchitis, unspecified
J21.0 Acute bronchiolitis due to respiratory syncytial virus
J21.1 Acute bronchiolitis due to human megapneumovirus
J21.8 Acute bronchiolitis due to other specified organisms
J21.9 Acute bronchiolitis, unspecified
J22 Unspecified acute lower respiratory infection
J22.0 (no description found)
J40 Bronchitis, not specified as acute or chronic
J41.0 Simple chronic bronchitis
J41.1 Mucopurulent chronic bronchitis
J41.8 Mixed simple and mucopurulent chronic bronchitis
J42 Unspecified chronic bronchitis
J44.0 Chronic obstructive pulmonary disease with acute lower respiratory infection
J47 Bronchiectasis
J65 Pneumoconiosis associated with tuberculosis
J85.0 Gangrene and necrosis of lung
J85.1 Abscess of lung with pneumonia
J85.2 Abscess of lung without pneumonia
J86.0 Pyothorax with fistula
J86.9 Pyothorax without fistula
P23.0 Congenital pneumonia due to viral agent
P23.2 Congenital pneumonia due to staphylococcus
P23.3 Congenital pneumonia due to staphylococcus, group B
P23.4 Congenital pneumonia due to Escherichia coli
P23.5 Congenital pneumonia due to Pseudomonas
P23.6 Congenital pneumonia due to other bacterial agents
P23.8 Congenital pneumonia due to other organisms
P23.9 Congenital pneumonia, unspecified

A31.1 Cutaneous mycobacterial infection
A46 Erysipelas
B00.0 Eczema herpeticum
B35.0 Tinea barbae and tinea capitis
B35.1 Tinea unguium
B35.2 Tinea manuum
B35.3 Tinea pedis
B35.4 Tinea corporis
B35.5 Tinea imbricate
B35.6 Tinea cruris
B35.8 Other dermatophytoses
B35.9 Dermatophytosis, unspecified
B36.0 Pityriasis versicolour
B36.1 Tinea nigra
B36.2 White piedra
B36.3 Black piedra
B36.8 Other specified superficial mycoses
B37.2 Candidiasis of skin and nail

B85.0 Pediculosis due to Pediculus humanus capitis
B85.1 Pediculosis due to Pediculus humanus corporis
B85.2 Pediculosis, unspecified
B85.3 Phthiriasis
B85.4 Mixed pediculosis and phthiriasis
B86 Scabies
B87.9 Myiasis, unspecified
B88.0 Other acariasis
B88.1 Tungiasis [sandflea infestation]
B88.2 Other arthropod infestations
B88.3 External hirudiniasis
B88.8 Other specified infestations
B88.9 Infestation, unspecified
H60.3 Other infective otitis externa
L00 Staphylococcal scalded skin syndrome
L01.0 Impetigo [any organism] [any site]
L02.0 Cutaneous abscess, furuncle and carbuncle of face
L02.1 Cutaneous abscess, furuncle and carbuncle of neck
L02.2 Cutaneous abscess, furuncle and carbuncle of trunk
L02.3 Cutaneous abscess, furuncle and carbuncle of buttock
L02.4 Cutaneous abscess, furuncle and carbuncle of limb
L02.8 Cutaneous abscess, furuncle and carbuncle of other sites
L02.9 Cutaneous abscess, furuncle and carbuncle, unspecified
L03.01 Cellulitis of finger
L03.02 Cellulitis of toe
L03.10 Cellulitis of upper limb
L03.11 Cellulitis of lower limb
L03.2 Cellulitis of face
L03.3 Cellulitis of trunk
L03.8 Cellulitis of other sites
L03.9 Cellulitis, unspecified
L05.0 Pilonidal cyst with abscess
L05.9 Pilonidal cyst without abscess
L08.0 Pyoderma
L08.1 Erythrasma
L08.8 Other specified local infections of skin and subcutaneous tissue
L08.9 Local infection of the skin and subcutaneous tissue, unspecified
L13.0 Dermatitis herpetiformis
L30.3 Infective dermatitis
L88 Pyoderma gangrenosum
M60.09 Infective myositis, multiple sites
M63.0 Myositis in bacterial diseases classified elsewhere
M63.1 Myositis in protozoal and parasitic infections classified elsewhere
M63.2 Myositis in other infectious diseases classified elsewhere
M65.0 Abscess of tendon sheath
M65.1 Other infective (teno)synovitis
M68.0 Synovitis and tenosynovitis in bacterial diseases classified elsewhere
M71.0 Abscess of bursa
M71.1 Other infective bursitis
O91.00 Infection of nipple associated with childbirth, without mention of attachment difficulty
O91.10 Abscess of breast associated with childbirth, without mention of attachment difficulty
P39.0 Neonatal infective mastitis
P39.4 Neonatal skin infection

A36.0 Pharyngeal diphtheria
A36.1 Nasopharyngeal diphtheria
A36.2 Laryngeal diphtheria
B05.3 Measles complicated by otitis media
H65.0 Acute serous otitis media
H65.1 Other acute nonsuppurative otitis media
H65.2 Chronic serous otitis media
H65.3 Chronic mucoid otitis media
H65.4 Other chronic nonsuppurative otitis media
H65.9 Nonsuppurative otitis media, unspecified
H66.0 Acute suppurative otitis media
H66.1 Chronic tubotympanic suppurative otitis media
H66.2 Chronic atticoantral suppurative otitis media
H66.3 Other chronic suppurative otitis media
H66.4 Suppurative otitis media, unspecified
H66.9 Otitis media, unspecified
H67.0 Otitis media in bacterial diseases classified elsewhere
H67.8 Otitis media in other diseases classified elsewhere
H68.0 Eustachian salpingitis
H70.0 Acute mastoiditis
H70.1 Chronic mastoiditis
H70.2 Petrositis
H70.8 Other mastoiditis and related conditions
H70.9 Mastoiditis, unspecified
H72.0 Central perforation of tympanic membrane
H72.1 Attic perforation of tympanic membrane
H72.2 Other marginal perforations of tympanic membrane
H72.8 Other perforations of tympanic membrane
H72.9 Perforation of tympanic membrane, unspecified
H73.0 Acute myringitis
H75.0 Mastoiditis in infectious and parasitic diseases classified elsewhere
H83.0 Labyrinthitis
H92.1 Otorrhoea
J00 Acute nasopharyngitis [common cold]
J01.0 Acute maxillary sinusitis
J01.1 Acute frontal sinusitis
J01.2 Acute ethmoidal sinusitis
J01.3 Acute sphenoidal sinusitis
J01.4 Acute pansinusitis
J01.8 Other acute sinusitis
J01.9 Acute sinusitis, unspecified
J02.0 Streptococcal pharyngitis
J02.8 Acute pharyngitis due to other specified organisms
J02.9 Acute pharyngitis, unspecified
J03.0 Streptococcal tonsillitis
J03.8 Acute tonsillitis due to other specified organisms
J03.9 Acute tonsillitis, unspecified
J04.0 Acute laryngitis
J04.1 Acute tracheitis
J04.2 Acute laryngotracheitis
J05.1 Acute epiglottitis
J06.0 Acute laryngopharyngitis
J06.8 Other acute upper respiratory infections of multiple sites
J06.9 Acute upper respiratory infection, unspecified

J32.0 Chronic maxillary sinusitis
J32.1 Chronic frontal sinusitis
J32.2 Chronic ethmoidal sinusitis
J32.3 Chronic sphenoidal sinusitis
J32.4 Chronic pansinusitis
J32.8 Other chronic sinusitis
J32.9 Chronic sinusitis, unspecified
J34.0 Abscess, furuncle and carbuncle of nose
J35.0 Chronic tonsillitis
J36 Peritonsillar abscess

A51.0 Primary genital syphilis
A51.1 Primary anal syphilis
A51.2 Primary syphilis of other sites
A51.3 Secondary syphilis of skin and mucous membranes
A51.4 Other secondary syphilis
A51.5 Early syphilis, latent
A51.9 Early syphilis, unspecified
A52.9 Late syphilis, unspecified
A53.0 Latent syphilis, unspecified as early or late
A53.9 Syphilis, unspecified
A54.0 Gonococcal infection of lower genitourinary tract without periurethral or accessory gland abscess
A54.1 Gonococcal infection of lower genitourinary tract with periurethral and accessory gland abscess
A54.2 Gonococcal pelviperitonitis and other gonococcal genitourinary infections
A54.3 Gonococcal infection of eye
A54.3 (no description found)
A54.4 Gonococcal infection of musculoskeletal system
A54.5 Gonococcal pharyngitis
A54.6 Gonococcal infection of anus and rectum
A54.8 Other gonococcal infections
A54.8 (no description found)
A54.9 Gonococcal infection, unspecified
A55 Chlamydial lymphogranuloma (venereum)
A56.0 Chlamydial infection of lower genitourinary tract
A56.1 Chlamydial infection of pelviperitoneum and other genitourinary organs
A56.2 Chlamydial infection of genitourinary tract, unspecified
A56.3 Chlamydial infection of anus and rectum
A56.4 Chlamydial infection of pharynx
A56.8 Sexually transmitted chlamydial infection of other sites
A57 Chancroid
A58 Granuloma inguinale
A59.0 Urogenital trichomoniasis
A59.0 (no description found)
A60.0 Herpesviral infection of genitalia and urogenital tract
A60.1 Herpesviral infection of perianal skin and rectum
A60.9 Anogenital herpesviral infection, unpsecified
A63.0 Anogenital (venereal) warts
A63.8 Other specified predominantly sexually transmitted diseases
A64 Unspecified sexually transmitted disease
B37.3 Candidiasis of vulva and vagina
B37.4 Candidiasis of other urogenital sites
N13.6 Pyonephrosis
N15.1 Renal and perinephric abscess
N30.0 Acute cystitis

N30.8 Other cystitis, abscess of bladder
N34.0 Urethral abscess
N35.1 Postinfective urethral stricture, not elsewhere classified
N39.0 Urinary tract infection, site not specified
N41.0 Acute prostatitis
N41.2 Abscess of prostate
N43.1 Infected hydrocele
N45.0 Orchitis, epididymitis and epididymo-orchitis with abscess
N45.9 Orchitis, epididymitis and epididymo-orchits without abscess
N51.2 Balanitis in diseases classified elsewhere
N70.0 Acute salpingitis and oophoritis
N73.0 Acute parametritis and pelvic cellulitis
N73.1 Chronic parametritis and pelvic cellulitis
N73.2 Unspecified parametritis and pelvic cellulitis
N73.3 Female acute pelvic peritonitis
N73.4 Female chronic pelvic peritonitis
N73.9 Female pelvic inflammatory disease, unspecified
N74.2 Female syphilitic pelvic inflammatory disease
N74.3 Female gonococcal pelvic inflammatory disease
N74.4 Female chlamydial pelvic inflammatory disease
N75.0 Cyst of Bartholins gland
N75.1 Abscess of Bartholins gland
N76.0 Acute vaginitis
N76.4 Abscess of vulva
O03.0 Spontaneous abortion, incomplete, complicated by genital tract and pelvic infection
O03.5 Spontaneous abortion, complete or unspecified, complicated by genital tract and pelvic infection
O08.0 Genital tract and pelvic infection following ectopic and molar pregnancy
O26.4 Herpes gestationis
O86.2 Urinary tract infection following delivery
O98.1 Syphilis complicating pregnancy, childbirth and the puerperium
O98.2 Gonorrhea complicating pregnancy, childbirth and the puerperium
P39.3 Neonatal urinary tract infection

A80.1 Acute paralytic poliomyelitis, wild virus, imported
A80.2 Acute paralytic poliomyelitis, wild virus, indigenous
A80.3 Acute paralytic poliomyelitis, other and unspecified
A80.4 Acute nonparalytic poliomyelitis
A80.9 Acute poliomyelitis, unspecified
A81.8 Other atypical virus infections of central nervous system
A81.9 Atypical virus infection of central nervous system, unspecified
A82.0 Sylvatic rabies
A82.1 Urban rabies
A82.9 Rabies, unspecified
A83.0 Japanese encephalitis
A83.1 Western equine encephalitis
A83.2 Eastern equine encephalitis
A83.3 St Louis encephalitis
A83.4 Australian encephalitis
A83.5 California encephalitis
A83.6 Rocio virus disease
A83.8 Other mosquito-borne viral encephalitis
A83.9 Mosquito-borne viral encephalitis, unspecified
A84.0 Far Eastern tick-borne encephalitis [Russian spring-summer encephalitis]
A84.1 Central European tick-borne encephalitis

A84.8 Other tick-borne viral encephalitis
A84.9 Tick-borne viral encephalitis, unspecified
A85.0 Enteroviral encephalitis
A85.1 Adenoviral encephalitis
A85.2 Arthropod-borne viral encephalitis, unspecified
A85.8 Other specified viral encephalitis
A86 Unspecified viral encephalitis
A87.0 Enteroviral meningitis
A87.1 Adenoviral meningitis
A87.2 Lymphocytic choriomeningitis
A87.8 Other viral meningitis
A87.9 Viral meningitis, unspecified
A88.0 Enteroviral exanthematous fever
A88.8 Other specified viral infections of central nervous system
A89 Unspecified viral infection of central nervous system
A90 Dengue fever [classical dengue]
A91 Dengue haemorrhagic fever
A92.0 Chikungunya virus disease
A92.1 O'nyong-nyong fever
A92.2 Venezuelan equine fever
A92.3 West Nile virus infection
A92.4 Rift Valley fever
A92.8 Other specified mosquito-borne viral fevers
A92.9 Mosquito-borne viral fever, unspecified
A93.0 Oropouche virus disease
A93.2 Colorado tick fever
A93.8 Other specified arthropod-borne viral fevers
A94 Unspecified arthropod-borne viral fever
A95.0 Sylvatic yellow fever
A95.1 Urban yellow fever
A95.9 Yellow fever, unspecified
A96.0 Junin haemorrhagic fever
A96.1 Machupo haemorrhagic fever
A96.2 Lassa fever
A96.8 Other arenaviral hemorrhagic fevers
A96.9 Arenaviral haemorrhagic fever, unspecified
A98.0 Crimean-Congo hemorrhagic fever
A98.1 Omsk hemorrhagic fever
A98.2 Kyasanur Forest disease
A98.3 Marburg virus disease
A98.4 Ebola virus disease
A98.5 Hemorrhagic fever with renal syndrome
A98.8 Other specified viral haemorrhagic fevers
A99 Unspecified viral haemorrhagic fever
B00.1 Herpesviral vesicular dermatitis
B00.2 Herpesviral gingivostomatitis and pharyngotonsillitis
B00.3 Herpesviral meningitis
B00.4 Herpesviral encephalitis
B00.5 Herpesviral ocular disease
B00.7 Disseminated herpesviral disease
B00.8 Other forms of herpesviral infection
B00.9 Herpesviral infection, unspecified
B01.0 Varicella meningitis
B01.1 Varicella encephalitis

B01.8 Varicella with other complications
B01.9 Varicella without complication
B02.0 Zoster encephalitis
B02.1 Zoster meningitis
B02.2 Zoster with other nervous system involvement
B02.3 Zoster ocular disease
B02.7 Disseminated zoster
B02.8 Zoster with other complications
B02.9 Zoster without complications
B03 Smallpox
B04 Monkeypox
B05.0 Measles complicated by encephalitis
B05.1 Measles complicated by meningitis
B05.4 Measles with intestinal complications
B05.8 Measles with other complications
B05.9 Measles without complication
B06.0 Rubella with neurological complications
B06.8 Rubella with other complications
B06.9 Rubella without complication
B07 Viral warts
B08.0 Other orthopoxvirus infections
B08.1 Molluscum contagiosum
B08.2 Exanthema subitum [sixth disease]
B08.3 Erythema infectiosum [fifth disease]
B08.4 Enteroviral vesicular stomatitis with exanthem
B08.5 Enteroviral vesicular pharyngitis
B08.8 Other specified viral infections characterized by skin and mucous membrane lesions
B09 Unspecified viral infection characterized by skin and mucous membrane lesions
B15.0 Hepatitis A with hepatic coma
B15.9 Hepatitis A without hepatic coma
B16.0 Acute hepatitis B with delta-agent with hepatic coma
B16.1 Acute hepatitis B with delta-agent without hepatic coma
B16.2 Acute hepatitis B without delta-agent with hepatic coma
B16.9 Acute hepatitis B without delta-agent and without hepatic coma
B17.0 Acute delta-(super) infection of hepatitis B carrier
B17.1 Acute hepatitis C
B17.2 Acute hepatitis E
B17.8 Other specified acute viral hepatitis
B17.9 Acute viral hepatitis, unspecified
B18.0 Chronic viral hepatitis B with delta-agent
B18.1 Chronic viral hepatitis B without delta-agent
B18.2 Chronic viral hepatitis C
B18.8 Other chronic viral hepatitis
B18.9 Chronic viral hepatitis, unspecified
B19.0 Unspecified viral hepatitis with hepatic coma
B19.9 Unspecified viral hepatitis without hepatic coma
B20.0 HIV disease resulting in mycobacterial infection
B20.1 HIV disease resulting in other bacterial infections
B20.2 HIV disease resulting in cytomegaloviral disease
B20.3 HIV disease resulting in other viral infections
B20.4 HIV disease resulting in candidiasis
B20.5 HIV disease resulting in other mycoses
B20.6 HIV disease resulting in Pneumocystis jirovecii pneumonia
B20.7 HIV disease resulting in multiple infections

B20.8 HIV disease resulting in other infectious and parasitic diseases
B20.9 HIV disease resulting in unspecified infectious or parasitic
B21.0 HIV disease resulting in Kaposi sarcoma
B21.1 HIV disease resulting in Burkitt lymphoma
B21.2 HIV disease resulting in other types of non-Hodgkin lymphoma
B21.3 HIV disease resulting in other malignant neoplasms of lymphoid, haematopoietic and related tissue
B21.7 HIV disease resulting in multiple malignant neoplasms
B21.8 HIV disease resulting in other malignant neoplasms
B21.9 HIV disease resulting in unspecified malignant neoplasm
B22.0 HIV disease resulting in encephalopathy
B22.1 HIV disease resulting in lymphoid interstitial pneumonitis
B22.2 HIV disease resulting in wasting syndrome
B22.7 HIV disease resulting in multiple diseases classified elsewhere
B23.0 Acute HIV infection syndrome
B23.1 HIV disease resulting in (persistent) generalized lymphadenopathy
B23.2 HIV disease resulting in haematological and immunological abnormalities, not elsewhere classified
B23.8 HIV disease resulting in other specified conditions
B24 Unspecified human immunodeficiency virus [HIV] disease
B25.0 Cytomegaloviral pneumonitis
B25.1 Cytomegaloviral hepatitis
B25.2 Cytomegaloviral pancreatitis
B25.8 Other cytomegaloviral diseases
B25.9 Cytomegaloviral disease, unspecified
B26.0 Mumps orchitis
B26.1 Mumps meningitis
B26.2 Mumps encephalitis
B26.3 Mumps pancreatitis
B26.8 Mumps with other complications
B26.8 (no description found)
B26.9 Mumps without complication
B27.0 Gammaherpesviral mononucleosis
B27.1 Cytomegaloviral mononucleosis
B27.8 Other infectious mononucleosis
B27.9 Infectious mononucleosis, unspecified
B30.0 Keratoconjunctivitis due to adenovirus
B30.1 Conjunctivitis due to adenovirus
B30.2 (no description found)
B30.3 Acute epidemic hemorrhagic conjunctivitis (enteroviral)
B30.8 Other viral conjunctivitis
B30.9 Viral conjunctivitis, unspecified
B33.0 Epidemic myalgia
B33.1 Ross River disease
B33.2 Viral carditis
B33.3 Retrovirus infections, not elsewhere classified
B33.4 Hantavirus (cardio-) pulmonary syndrome
B33.8 Other specified viral diseases
B34.1 Enterovirus infection, unspecified
B34.2 Coronavirus infection, unspecified site
B34.3 Parvovirus infection, unspecified site
B34.4 Papovavirus infection, unspecified
B34.8 Other viral infections of unspecified site
B34.9 Viral infection, unspecified
B97.0 Adenovirus as the cause of diseases classified to other chapters
B97.1 Enterovirus as the cause of diseases classified to other chapters

B97.2 Coronavirus as the cause of diseases classified to other chapters
B97.3 Retrovirus as the cause of diseases classified to other chapters
B97.4 Respiratory syncytial virus as the cause of diseases classified to other chapters
B97.5 Reovirus as the cause of diseases classified to other chapters
B97.6 Parvovirus as the cause of diseases classified to other chapters
B97.7 Papillomavirus as the cause of diseases classified to other chapters
B97.8 Other viral agents as the cause of diseases classified to other chapters
G02.0 Meningitis in viral diseases classified elsewhere
G05.1 Encephalitis, myelitis and encephalomyelitis in viral diseases classified elsewhere
H19.1 Herpesviral keratitis and keratoconjunctivitis
I41.1 Myocarditis in viral diseases classified elsewhere
J10.8 Influenza with other manifestations, influenza virus identified
J11 Influenza, virus not identified
J11.8 Influenza with other manifestations, virus not identified
M01.4 Rubella arthritis
M01.50 Arthritis in other viral diseases classified elsewhere, multiple sites
M01.51 Arthritis in other viral diseases classified elsewhere, shoulder region
M01.52 Arthritis in other viral diseases classified elsewhere, upper arm
M01.53 Arthritis in other viral diseases classified elsewhere, forearm
M01.54 Arthritis in other viral diseases classified elsewhere, hand
M01.55 Arthritis in other viral diseases classified elsewhere, pelvic region and thigh
M01.56 Arthritis in other viral diseases classified elsewhere, lower leg
M01.57 Arthritis in other viral diseases classified elsewhere, ankle and foot
M01.58 Arthritis in other viral diseases classified elsewhere, other site
M01.59 Arthritis in other viral diseases classified elsewhere, site unspecified
O98.4 Viral hepatitis complicating pregnancy, childbirth and the puerperium
O98.5 Other viral diseases complicating pregnancy, childbirth and the puerperium
P35.0 Congenital rubella syndrome
P35.1 Congenital cytomegalovirus infection
P35.2 Congenital herpesviral [herpes simples] infection
P35.3 Congenital viral hepatitis
P35.8 Other congenital viral diseases
P35.9 Congenital viral disease, unspecified
Z21 Asymptomatic human immunodeficiency virus [HIV] infection status

Respiratory non-infection

J30-J309	Vasomotor and allergic rhinitis
J31-J312	Chronic rhinitis, nasopharyngitis and pharyngitis
J32-J329	Chronic sinusitis
J33-J339	Nasal polyp
J34-J349	Other and unsepcified disorders of nose and nasal sinuses
J3501-J359	Chronic diseases of tonsils and adenoids
J36	Peritonsillar abscess
J37-J371	Chronic laryngitis and laryngotracheitis
J38-J387	Diseases of vocal cords and larynx, not elsewhere classified
J39-J399	Other diseases of upper respiratory tract
J40	Bronchitis, not specified as acute or chronic
J41	Simple and mucopurulent chronic bronchitis
J42	Unspecified chronic bronchitis
J43	Emphysema
J44	Other chronic obstructive pulmonary disease
J45	Asthma
J47	Bronchiectasis
J80	Acute respiratory distress syndrome
J81	Pulmonary edema
J82	Pulmonary eosinophilia, not elsewhere classified
J84	Other interstitial pulmonary diseases
J85	Abscess of lung and mediastinum
J86	Pyothorax
J90	Pleural effusion, not elsewhere classified
J91	Pleural effusion in conditions classified elsewhere
J92	Pleural plaque
J93	Pneumothorax and air leak
J94	Other pleural conditions
J95	Intraoperative and postprocedural complications and disorders of respiratory system, not elsewhere classified
J96	Respiratory failure, not elsewhere classified
J98	Other respiratory disorders
J99	Respiratory disorders in diseases classified elsewhere

Exclude:

J849	Interstitial pulmonary disease, unspecified

Gastrointestinal non-infection

K20	Esophagitis
K21	Gastro-esophageal reflux disease
K22	Other diseases of esophagus
K23	Disorders of esophagus in diseases classified elsewhere
K25	Gastric ulcer
K26	Duodenal ulcer
K27	Peptic ulcer, site unspecified
K28	Gastrojejunal ulcer
K29	Gastritis and duodenitis
K30	Functional dyspepsia
K31	Other diseases of stomach and duodenum
K90	Intestinal malabsorption
K91	Intraoperative and postprocedural complications and disorders of digestive system, not elsewhere classified
K92	Other diseases of digestive system
K40	Inguinal hernia
K41	Femoral hernia
K42	Umbilical hernia
K43	Ventral hernia
K44	Diaphragmatic hernia
K45	Other abdominal hernia
K46	Unspecified abdominal hernia
K50	Crohn's disease
K51	Ulcerative colitis
K52	Other and unspecified noninfective gastroenteritis and colitis
K55	Vascular disorders of intestine
K56	Paralytic ileus and intestinal obstruction without hernia
K57	Diverticular disease of intestine
K58	Irritable bowel syndrome
K59	Other functional intestinal disorders
K60	Fissure and fistula of anal and rectal regions
K61	Abcess of anal and rectal regions
K62	Other diseases of anus and rectum
K63	Other diseases of intestine
K65	Peritonitis
K66	Other disorders of peritoneum

Exclude:

K650	Generalized (acute) peritonitis
K651	Peritoneal abscess
K652	Spontaneous bacterial peritonitis

Oral cavity

K00-K09	Diseases of oral cavity and salivary glands
K01	Dysphagia
K02-K029	Dental caries
K03-K039	Other diseases of hard tissues of teeth
K04-K0499	Diseases of pulp and periapical tissues
K05-K056	Gingivitis and periodontal diseases
K06-K069	Other disorders of gingiva and eentulous alveolar ridge
K08-K089	Other disorders of teeth and supporting structures
K09-K099	Cysts of oral region, not elsewhere classified
K11-K119	Diseases of salivary glands
K12-K1239	Stomatitis and related lesions
K13-K1379	Other diseases of lip and oral mucosa
K14-K149	Diseases of tongue
R1310	Dysphagia
Z012	Encounter for dental examination and cleaning
Z463	Encounter for fitting and adjustment of dental prosthetic device
Z464	Encounter for fitting and adjustment of orthodontic device

Exclude:

K0520	Aggressive periodontitis
K0521	Aggressive periodontitis, localized
K0522	Aggressive periodontitis, generalized

Congenital anomalies

Q00-Q07	Congenital malformations of the nervous system
Q10-Q18	Congenital malformations of eye, ear, face and neck
Q20-Q28	Congenital malformations of the circulatory system
Q30-Q34	Congenital malformations of the respiratory system
Q35-Q37	Cleft lip and cleft palate
Q38-Q45	Other congenital malformations of the digestive system
Q50-Q56	Congenital malformations of genital organs
Q60-Q64	Congenital malformations of the urinary system
Q65-Q79	Congenital malformations and deformations of the musculoskeletal system
Q80-Q89	Other congenital malformations
Q90-Q99	Chromosomal abnormalities, not elsewhere classified

Social

Z55-Z65	Person with potential health hazards related to socio-economic and psychological circumstances
Z742-Z749	Care provider dependency
Z761	Supervision and care of foundling
Z762	Supervision and care of health infant/child
Z0441-Z0442	Examination following suspected rape
Z0471-Z0472	Examination following suspected physical abuse

Exclude:

Z57-Z579	Health issues due to occupational exposures
Z64	Psychological problems related to unwanted pregnancy/multiparity
Z56-Z569	Health issues due to unemployment

Mental health

F90-F909	Attention-deficit hyperactivity disorders
F91-F919	Conduct disorders
F93-F939	Emotional disorders with onset specific to childhood
F94-F949	Disorders of social functioning with onset specific to childhood and adolescence
F95-F959	Tic disorder
F98-F989	Other behavioral and emotional disorders with onset usually occurring in childhood and adolescence
F99	Mental disorder, not otherwise specified
F40-F409	Phobic anxiety disorders
F41-F419	Other anxiety disorders
F422-F429	Obsessive-compulsive disorder
F43-F439	Reaction to severe stress, and adjustment disorders
F481-F489	Other nonpsychotic mental disorders

Injury/external

J60	Coalworker's pneumoconiosis
J61	Pneumoconiosis due to asbestos and other mineral fibres
J62	Pneumoconiosis due to dust containing silica
J63	Pneumoconiosis due to other inorganic dusts
J64	Unspecified pneumoconiosis
J65	Pneumoconiosis associated with tuberculosis
J66	Airway disease due to specific organic dust
J67	Hypersensitivity pneumonitis due to organic dust
J68	Respiratory conditions due to inhalation of chemicals, gases, fumes and vapours
J69	Pneumonitis due to solids and liquids
J70	Respiratory conditions due to other external agents
S00-S09	Injuries to the head
S10-S19	Injuries to the neck
S20-S29	Injuries to the thorax
S30-S39	Injuries to the abdomen, lower back, lumbar spine and pelvis
S40-S49	Injuries to the shoulder and upper arm
S50-S59	Injuries to the elbow and forearm
S60-S69	Injuries to the wrist and hand
S70-S79	Injuries to the hip and thigh
S80-S89	Injuries to the knee and lower leg
S90-S99	Injuries to the ankle and foot
T00-T07	Injuries involving multiple body regions
T08-T14	Injuries to unspecified parts of trunk, limb or body region
T15-T19	Effects of foreign body entering through natural orifice
T20-T32	Burns and corrosions
T33-T35	Frostbite
T36-T50	Poisoning by drugs, medicaments and biological substances
T51-T65	Toxic effects of substances chiefly nonmedicinal as to source
T66-T78	Other and unspecified effects of external causes
T79	Certain early complications of trauma
T80-T88	Complications of surgical and medical care, not elsewhere classified
T90-T98	Sequelae of injuries, of poisoning and of other consequences of external causes
P10-P15	Birth trauma
V01-V99	Transport accidents
W00-X59	Other external causes of accidental injury
Y85-Y89	Sequelae of external causes of morbidity and mortality

Renal/genitourinary

N00	Acute nephritic syndrome
N01	Rapidly progressive nephritic syndrome
N02	Recurrent and persistent haematuria
N03	Chronic nephritic syndrome
N04	Nephrotic syndrome
N05	Unspecified nephritic syndrome
N06	Isolated proteinuria with specified morphological lesion
N07	Hereditary nephropathy, not elsewhere classified
N08	Glomerular disorders in diseases classified elsewhere
N10	Acute tubulo-interstitial nephritis
N11	Chronic tubulo-interstitial nephritis
N12	Tubulo-interstitial nephritis, not specified as acute or chronic
N13	Obstructive and reflux uropathy
N14	Drug- and heavy-metal-induced tubulo-interstitial and tubular conditions
N15	Other renal tubulo-interstitial diseases
N16	Renal tubulo-interstitial disorders in diseases classified elsewhere
N17	Acute renal failure
N18	Chronic renal failure
N19	Unspecified renal failure
N20	Calculus of kidney and ureter
N21	Calculus of lower urinary tract
N22	Calculus of urinary tract in diseases classified elsewhere
N23	Unspecified renal colic
N25	Disorders resulting from impaired renal tubular function
N26	Unspecified contracted kidney
N27	Small kidney of unknown cause
N28	Other disorders of kidney and ureter, not elsewhere classified
N29	Other disorders of kidney and ureter in diseases classified elsewhere
N30	Cystitis
N31	Neuromuscular dysfunction of bladder, not elsewhere classified
N32	Other disorders of bladder
N33	Bladder disorders in diseases classified elsewhere
N34	Urethritis and urethral syndrome
N35	Urethral stricture
N36	Other disorders of urethra
N37	Urethral disorders in diseases classified elsewhere
N39	Other disorders of urinary system
N40	Hyperplasia of prostate
N41	Inflammatory diseases of prostate
N42	Other disorders of prostate
N43	Hydrocele and spermatocele
N44	Torsion of testis
N45	Orchitis and epididymitis
N46	Male infertility
N47	Redundant prepuce, phimosis and paraphimosis
N48	Other disorders of penis
N49	Inflammatory disorders of male genital organs, not elsewhere classified
N50	Other disorders of male genital organs
N51	Disorders of male genital organs in diseases classified elsewhere
N60	Benign mammary dysplasia
N61	Inflammatory disorders of breast
N62	Hypertrophy of breast

N63	Unspecified lump in breast
N64	Other disorders of breast
N70	Salpingitis and oophoritis
N71	Inflammatory disease of uterus, except cervix
N72	Inflammatory disease of cervix uteri
N73	Other female pelvic inflammatory diseases
N74	Female pelvic inflammatory disorders in diseases classified elsewhere
N75	Diseases of Bartholin's gland
N76	Other inflammation of vagina and vulva
N77	Vulvovaginal ulceration and inflammation in diseases classified elsewhere
N80	Endometriosis
N81	Female genital prolapse
N82	Fistulae involving female genital tract
N83	Noninflammatory disorders of ovary, fallopian tube and broad ligament
N84	Polyp of female genital tract
N85	Other noninflammatory disorders of uterus, except cervix
N86	Erosion and ectropion of cervix uteri
N87	Dysplasia of cervix uteri
N88	Other noninflammatory disorders of cervix uteri
N89	Other noninflammatory disorders of vagina
N90	Other noninflammatory disorders of vulva and perineum
N91	Absent, scanty and rare menstruation
N92	Excessive, frequent and irregular menstruation
N93	Other abnormal uterine and vaginal bleeding
N94	Pain and other conditions associated with female genital organs and menstrual cycle

Exclude:

N2883	Nephroptosis
N1370	Vesicoureteral-reflux
N1372	Vesicoureteral-reflux with reflux nephropathy without hydroureter

<u>Neoplasms</u>

C00-C96Z	Malignant neoplasm
D00-D099	In situ neoplasm
D10-D369	Benign neoplasm
D3701-D489	Neoplasm of uncertain behaviour
D3A00-D3A8	Benign tumours
D49-D499	Neoplasms of uncertain or unknown behaviour

Exclude:

D18	Hemangioma and lymphangioma, any site

Central nervous system

G03 Meningitis due to other and unspecified causes
G04 Encephalitis, myelitis and encephalomyelitis
G08 Intracranial and intraspinal phlebitis and thrombophlebitis
G09 Sequelae of inflammatory diseases of central nervous system
G10 Huntington's disease
G11 Hereditary ataxia
G12 Spinal muscular atrophy and related syndromes
G13 Systemic atrophies primarily affecting central nervous system in diseases classified elsewhere
G20 Parkinson's disease
G21 Secondary parkinsonism
G22 Parkinsonism in diseases classified elsewhere
G23 Other degenerative diseases of basal ganglia
G24 Dystonia
G25 Other extrapyramidal and movement disorders
G26 Extrapyramidal and movement disorders in diseases classified elsewhere
G30 Alzheimer's disease
G31 Other degenerative diseases of nervous system, not elsewhere classified
G32 Other degenerative disorders of nervous system in diseases classified elsewhere
G35 Multiple sclerosis
G36 Other acute disseminated demyelination
G37 Other demyelinating diseases of central nervous system
G40 Epilepsy
G41 Status epilepticus
G42 Migraine
G43 Other headache syndromes
G44 Transient cerebral ischaemic attacks and related syndromes
G45 Vascular syndromes of brain in cerebrovascular diseases
G46 Sleep disorders
G50 Disorders of trigeminal nerve
G51 Facial nerve disorders
G52 Disorders of other cranial nerves
G53 Cranial nerve disorders in diseases classified elsewhere
G54 Nerve root and plexus disorders
G55 Nerve root and plexus compressions in diseases classified elsewhere
G56 Mononeuropathies of upper limb
G57 Mononeuropathies of lower limb
G58 Other mononeuropathies
G59 Mononeuropathy in diseases classified elsewhere
G60 Hereditary and idiopathic neuropathy
G61 Inflammatory polyneuropathy
G62 Other polyneuropathies
G63 Polyneuropathy in diseases classified elsewhere
G64 Other disorders of peripheral nervous system
G70 Myasthenia gravis and other myoneural disorders
G71 Primary disorders of muscles
G72 Other myopathies
G73 Disorders of myoneural junction and muscle in diseases classified elsewhere
G80 Cerebral palsy
G81 Hemiplegia
G82 Paraplegia and tetraplegia
G83 Other paralytic syndromes
G90 Disorders of autonomic nervous system

G91	Hydrocephalus
G92	Toxic encephalopathy
G93	Other disorders of brain
G94	Other disorders of brain in diseases classified elsewhere
G95	Other diseases of spinal cord
G96	Other disorders of central nervous system
G97	Postprocedural disorders of nervous system, not elsewhere classified
G98	Other disorders of nervous system, not elsewhere classified
G99	Other disorders of nervous system in diseases classified elsewhere

Exclude:

G120	Infantile spinal muscular atrophy, type I, Werdnig-Hoffman
G610	Guillain-Barre syndrome
G712	Congenital myopathies
G710	Muscular dystrophy
G7111	Myotonic muscular dystrophy
G7112	Myotonia congenita
G7113	Myotonic chondrodystrophy
G7114	Drug induced myotonia
G7119	Other specified myotonic disorders
G723	Periodic paralysis

Perinatal

P00	Fetus and newborn affected by maternal conditions that may be unrelated to present pregnancy
P01	Fetus and newborn affected by maternal complications of pregnancy
P02	Fetus and newborn affected by complications of placenta, cord and membranes
P03	Fetus and newborn affected by other complications of labour and delivery
P04	Fetus and newborn affected by noxious influences transmitted via placenta or breast milk
P05	Slow fetal growth and fetal malnutrition
P07	Disorders related to short gestation and low birth weight, not elsewhere classified
P08	Disorders related to long gestation and high birth weight
P20	Intrauterine hypoxia
P21	Birth asphyxia
P24	Neonatal aspiration syndromes
P25	Interstitial emphysema and related conditions originating in the perinatal period
P26	Pulmonary haemorrhage originating in the perinatal period
P29	Cardiovascular disorders originating in the perinatal period
P50	Fetal blood loss
P51	Umbilical haemorrhage of newborn
P52	Intracranial nontraumatic haemorrhage of fetus and newborn
P53	Haemorrhagic disease of fetus and newborn
P54	Other neonatal haemorrhages
P55	Haemolytic disease of fetus and newborn
P56	Hydrops fetalis due to haemolytic disease
P57	Kernicterus
P58	Neonatal jaundice due to other excessive haemolysis
P59	Neonatal jaundice from other and unspecified causes
P60	Disseminated intravascular coagulation of fetus and newborn
P61	Other perinatal haematological disorders
P70	Transitory disorders of carbohydrate metabolism specific to fetus and newborn
P71	Transitory neonatal disorders of calcium and magnesium metabolism
P72	Other transitory neonatal endocrine disorders
P74	Other transitory neonatal electrolyte and metabolic disturbances
P75	Meconium ileus
P76	Other intestinal obstruction of newborn
P77	Necrotizing enterocolitis of fetus and newborn
P78	Other perinatal digestive system disorders
P80	Hypothermia of newborn
P81	Other disturbances of temperature regulation of newborn
P83	Other conditions of integument specific to fetus and newborn
P90	Convulsions of newborn
P91	Other disturbances of cerebral status of newborn
P92	Feeding problems of newborn
P93	Reactions and intoxications due to drugs administered to fetus and newborn
P94	Disorders of muscle tone of newborn
P95	Fetal death of unspecified cause
P96	Other conditions originating in the perinatal period
