# Supplementary material for: Gestational age and hospital admissions during childhood: population based, record linkage study in England (TIGAR study)
Source: BMJ. 2020 Nov 25;371:m4075. doi: 10.1136/bmj.m4075 (PMC7687266; doi:10.1136/bmj.m4075)
Supplement: Supplementary file 2 — Web appendix: Supplementary information [file coav055130.ww2.pdf]

Supplementary information

A. Quality assurance and data cleaning

Identifying duplicate episodes within HES:

1. Unfinished episodes were identified using the episode type variable (EPITYPE==1) and then excluded.

2. Duplicate HES birth records were identified by comparing HES ID, baby's DOB, Sex, mother's DOB, postcode, hospital location code, episode start and end dates, episode order, diagnosis and operation codes and episode key (EPIKEY). If all variables exactly match, any episode was selected and the remaining record/s were excluded.

3. Further duplicate HES birth records were identified by comparing HES ID, baby's DOB, Sex, mother's DOB, postcode, hospital location code, episode start and end dates, episode order, diagnosis and operation codes. If all variables exactly match, any episode was selected and the remaining record/s were excluded.

4. Multiple episodes were identified by comparing key characteristics of episodes. If HES ID, baby's DOB and sex matched and if mother's DOB, postcode and birthweight were either matching or had missing data, this was considered the same baby with multiple episodes within the birth spell.

5. The remaining cases were manually reviewed. In cases where two or more key variables (baby's DOB, Sex, mother's DOB, postcode, hospital location code) were different (not because one was missing), both records were excluded, as it was not clear which characteristics were correct.

Birth registration records linked to more than one HES birth record:

1. Key characteristics (baby's DOB, gestational age, birth weight, sex, mother's DOB and postcode) were compared and the record with the highest number of matching variables was identified as the correct link.

2. If records matched on the same number of variables, the record that matched exactly on birthweight was identified as the correct match.

3. If birthweight was missing or didn't match, then the record with the highest match rank score was identified as the correct link.

4. If records had the same match rank score, then both records were excluded, as it was not possible to identify the correct match.

B. Definitions of birth and hospital admissions

HES inpatient admissions are structured as ‘episodes’ of care, with an episode defined as a period of care under one consultant, within one hospital. Multiple episodes are referred to as a ‘spell’ and represent an uninterrupted period of care within one hospital. A new spell is generated when the patient is transferred to another hospital for continued care. A continuous inpatient stay (CIP) may consist of one episode or multiple episodes and spells, and ends when the patient is discharged from an NHS hospital. Hospital episodes are primarily collected for financial reimbursement, and therefore, the datasets are divided into financial years, beginning 1st April and ending 31st March. Episodes are labelled as ‘finished’ once the patient is discharged from hospital. However, if an episode begins in one financial year and ends during the next, two episodes will be generated – one in the financial year the episode begins and one in the financial year that the episode ends – therefore, the first episode will remain as ‘unfinished’.

C. Identifying hospital transfers

HES variables:

Admission method (ADMIMETH)

Admission source (ADMISORC)

Discharge destination (DISDEST)

Hospital admission defined as a transfer if:

1. first episode in spell

   AND

2. ADMIMETH = 81

OR

3. ADMISORC = 49, 50, 51, 52, 53 or 87

OR

4. DISDEST = 49, 50, 51, 52, 53 or 84

AND

5. ≤2 days between admission and discharge dates of previous/next spell

D. Identifying type of hospital admission:

Hospital admission is defined as an elective/planned admission if:

ADMIMETH:

11 = Waiting list

12 = Booked

13 = Planned

Hospital admission is defined as emergency admission if:

ADMIMETH:

21 = Accident & Emergency or dental casualty department of health care provider

22 = General Practitioner request for immediate admission

23 = Bed bureau

24 = Consultant clinic, of this or other health care provider

25 = Admission via mental health crisis resolution team

2A = Accident & emergency department of another provider where patient not admitted

2B = Transfer of admitted patient from another hospital provider

2C = Baby born at home as intend2D = Other emergency admission

28 = Other mean

Table S1. Data sources for key ONS variables to compare quality of linkage to HES

Variable	Source	Rule
Baby's date of birth	BR & BN	Use BR unless BR = missing, then use BN
Mother's date of birth	BR & BN	Use BR unless BR = missing, then use BN
Birth weight	BR & BN	Use BR unless BR = missing, then use BN
Gestational age	BR & BN	Use BN as BR reported for still births only
Sex	BR & BN	Use BR unless BR = missing, then use BN
Hospital of birth	BR & BN	Use BR unless BR = missing, then use BN

BR = Birth Registration, BN = Birth Notification (NN4B)

Table S2. Algorithm to quality assure linkage between Birth registration/Birth notification records and HES records

	Variable												
**Step**	**Hospital Trust code**		**DOB**		**Sex**		**Birthweight**		**Gestational age**		**Mother's DOB**		**Postcode**
1	E	*and*	E	*and*	E	*and*	E	*and*	E		-		-
2	E	*and*	E	*and*	E	*and*	(E	*or*	E)		-		-
3	E	*and*	E	*and*	E		-		-		-		-
4	-		E	*and*	E	*and*	E/M	*and*	E/M	*and*	E/M	*and*	E/M
5	E	*and*	P	*and*	E	*and*	(E	*or*	E	*or*	E	*or*	E)
6	E	*and*	E		-		E/M	*and*	E/M	*and*	E/M	*and*	E/M

E = Exact match; M = Missing; P = Partial match (differs by up to four days, two elements of data match or dates match if day and month swapped

E. High risk children

A child was defined as ‘high-risk’ if they had at least one of the following ICD10 diagnosis codes within their birth admission or subsequent readmission records:

Table S3. Definition of high-risk children

ICD10 code	Description	Record searched
D80	Immunodeficiency with predominantly antibody defects	Birth admission and all readmissions
D81	Combined immunodeficiency	Birth admission and all readmissions
D82	Immunodeficiency associated with other major defects	Birth admission and all readmissions
D83	Common variable immunodeficiency	Birth admission and all readmissions
D84	Other immunodeficiency	Birth admission and all readmissions
D89	Other disorders involving the immune mechanism	Birth admission and all readmissions
E84	Cystic fibrosis	Birth admission and all readmissions
Q	Congenital malformations	All birth and readmission records within the first year*
N18	Chronic kidney disease	Birth admission and all readmissions
D57	Sickle cell disease	Birth admission and all readmissions
C	Malignant neoplasm	Birth admission and all readmissions
D56·1	Beta-thalassemia	Birth admission and all readmissions

*Hospital records within the first year were searched to avoid including minor congenital anomalies that are only diagnosed later in childhood

F. Parity

Parity was available from two sources:

1. Birth registration
2. HES APC

However, both sources had quality issues. On the birth registration form, parity was only recorded for women who were married until May 2012. This means, missingness is due to being unmarried. In HES, between 2005 and 2006, some hospitals did not record parity accurately, with some recording all women as either nulliparous or parous during that year. Therefore, both sources were combined and birth registration parity was the preferred source for women who were married. To evaluate the likelihood that a hospital was reporting parity accurately, the proportion of nulliparous women in each hospital by year was calculated. Those hospitals reporting <20 or >70% of women as nulliparous were flagged as potentially inaccurately reporting parity. Those that were flagged were then excluded as part of a sensitivity analysis.

Table S4. Total number of hospital admissions experienced by children, according to gestational age

	0		1		2		3		4		5+	
**Gestational age (weeks)**	n	%	n	%	n	%	n	%	n	%	n	%
<28	103	6.0	295	17·1	282	16·3	187	10·8	167	9·7	696	40·2
28-29	263	12·6	467	22·4	348	16·7	262	12·5	183	8·8	566	27·1
30-31	590	18·3	840	26·0	582	18·0	336	10·4	233	7·2	646	20·0
32	637	24.0	699	26·3	440	16·6	263	9·9	177	6·7	440	16·6
33	1,035	25·6	1,104	27·3	695	17·2	453	11·2	229	5·7	534	13·2
34	2,225	30·5	2,050	28·1	1,182	16·2	653	9.0	384	5·3	798	10·9
35	4,051	34·7	3,225	27·7	1,802	15·5	994	8·5	528	4·5	1,063	9·1
36	8,822	37·8	6,272	26·9	3,456	14·8	1,824	7·8	1,007	4·3	1,965	8·4
37	22,830	42·3	14,153	26·2	7,421	13·7	3,753	7.0	2,036	3·8	3,808	7·1
38	64,098	46·5	35,897	26·0	17,174	12·5	8,661	6·3	4,453	3·2	7,643	5·5
39	114,209	49·4	59,465	25·7	27,649	12.0	13,155	5·7	6,581	2·8	10,317	4·5
40	145,809	50·6	74,001	25·7	33,725	11·7	15,415	5·4	7,717	2·7	11,398	4.0
41	106,847	51·2	53,423	25·6	24,003	11·5	11,186	5·4	5,261	2·5	8,037	3·9
42	21,580	51·4	10,715	25·5	4,824	11·5	2,131	5·1	1,064	2·5	1,644	3·9
**Overall**	493,099	48·4	262,606	25·8	123,583	12·2	59,273	5·8	30,020	3.0	49,555	4·9

Table S5. Adjusted rate ratios (RR) and 95% CI for hospital admissions during childhood by gestational age for each sensitivity analysis

Gestational age	Model 1 (n=712,801)			Model 2 (n=893,662)			Model 3 (n=578,448)			Model 4 (n=872,803)			Model 5 (n=793,945)		
	RR	95% CI		RR	95% CI		RR	95% CI		RR	95% CI		RR	95% CI	
<28	4·89	4·54	5·27	4·81	4·48	5·15	4·81	4·37	5·28	4·84	4·49	5·21	4.82	4.47	5.20
28-29	3·16	2·92	3·43	3·37	3·08	3·69	3·08	2·75	3·45	3·24	2·96	3·55	3.20	2.90	3.52
30-31	2·83	2·24	3·57	2·73	2·38	3·12	2·76	2·13	3·57	2·67	2·22	3·20	2.70	2.21	3.29
32	2·34	2·13	2·57	2·48	2·24	2·74	2·17	1·98	2·38	2·46	2·16	2·79	2.48	2.16	2.84
33	1·92	1·79	2·05	2·03	1·91	2·15	1·87	1·74	2·02	1·94	1·82	2·06	1.90	1.78	2.03
34	1·76	1·66	1·87	1·83	1·73	1·93	1·86	1·73	2·01	1·81	1·71	1·92	1.77	1.67	1.89
35	1·55	1·48	1·62	1·59	1·53	1·66	1·57	1·49	1·65	1·56	1·49	1·63	1.55	1.48	1.62
36	1·52	1·45	1·59	1·61	1·54	1·68	1·53	1·47	1·60	1·54	1·49	1·60	1.55	1.48	1.63
37	1·36	1·32	1·41	1·42	1·38	1·46	1·40	1·35	1·45	1·39	1·35	1·43	1.38	1.34	1.42
38	1·18	1·15	1·21	1·22	1·20	1·25	1·19	1·15	1·22	1·19	1·16	1·21	1.18	1.15	1.21
39	1·07	1·05	1·10	1·07	1·06	1·09	1·06	1·04	1·09	1·06	1·04	1·08	1.06	1.04	1.08
40	ref			ref			ref			ref			ref		
41	0·97	0·94	0·99	0·98	0·96	1·00	0·97	0·95	1·00	0·98	0·96	1·01	0.98	0.96	1.00
42	0·91	0·87	0·94	0·98	0·95	1·01	0·98	0·94	1·02	0·97	0·94	1·00	0.98	0.94	1.01

Model 1: Fully adjusted model + method of induction
Model 2: Fully adjusted model + birth admission length of stay
Model 3: Fully adjusted model excluding unreliable reporters of parity
Model 4: Fully adjusted model excluding second/third babies born during study period
Model 5: Fully adjusted model excluding SGA births

Table S6. Adjusted rate ratios (RR) and 95% confidence intervals (CI) of hospital admissions during child stratified by age at admission excluding high-risk group*

	<1 year (n= 814,852)			1-2 years (n=814,172)			3-4 years (n=813,917)			5-6 years (n=813,821)			7-10 years (n=813,774)		
	RR	95% CI		RR	95% CI		RR	95% CI		RR	95% CI		RR	95% CI	
Gestational age															
<28	5·22	4·59	5·95	3·50	3·01	4·06	2·63	2·20	3·14	1·95	1·59	2·39	1·68	1·32	2·15
28-29	4·26	3·87	4·69	2·74	2·44	3·06	2·27	1·99	2·59	2·16	1·87	2·50	2·10	1·78	2·48
30-31	3·24	3·01	3·48	2·12	1·95	2·31	1·88	1·70	2·08	1·66	1·48	1·85	1·54	1·36	1·76
32	2·65	2·45	2·88	1·97	1·79	2·15	1·74	1·56	1·94	1·67	1·48	1·87	1·62	1·42	1·86
33	2·34	2·19	2·50	1·76	1·63	1·89	1·55	1·42	1·70	1·53	1·39	1·69	1·63	1·46	1·82
34	2·18	2·07	2·29	1·56	1·47	1·65	1·32	1·23	1·41	1·26	1·16	1·36	1·22	1·11	1·33
35	1·89	1·82	1·98	1·44	1·37	1·51	1·30	1·23	1·37	1·29	1·22	1·37	1·25	1·17	1·34
36	1·84	1·78	1·89	1·31	1·26	1·35	1·22	1·17	1·27	1·18	1·13	1·23	1·18	1·12	1·24
37	1·58	1·54	1·61	1·22	1·19	1·25	1·19	1·15	1·22	1·19	1·16	1·23	1·25	1·21	1·29
38	1·26	1·24	1·28	1·12	1·10	1·14	1·08	1·06	1·10	1·08	1·05	1·10	1·07	1·04	1·10
39	1·10	1·08	1·11	1·04	1·02	1·05	1·03	1·02	1·05	1·03	1·01	1·05	1·01	0·99	1·03
40		ref			ref			ref			ref			ref	
41	0·93	0·92	0·95	0·98	0·96	0·99	1·01	0·99	1·02	1·00	0·98	1·02	1·00	0·98	1·02
42	0·90	0·87	0·92	0·97	0·94	0·99	0·98	0·95	1·01	0·98	0·95	1·02	0·99	0·95	1·03

* High-risk children defined as a child with diagnosis of malignant neoplasm, blood disorder, cystic fibrosis, immune dysfunction or congenital anomaly

Table S7. Crude rates per 100 person years for causes of admission, by gestational age at birth and age at admission

Gestational age	Infection	Perinatal	Congenital anomaly	Injury/external	CNS	Renal/GUI	GI tract	Respiratory	Oral cavity
**<1 year**									
<28	103	17	9	9	5	1	37	34	1
28-31	55	5	6	6	2	1	24	14	0
32-33	35	5	5	4	1	1	15	7	0
34-36	21	8	4	3	1	1	9	4	0
37-38	15	5	3	2	0	0	5	2	0
39-42	12	2	2	2	0	0	3	2	0
**1-2 years**									
	Infection	Perinatal	Congenital anomaly	Injury/external	CNS	Renal/GUI	GI tract	Respiratory	Oral cavity
<28	37	2	7	5	4	1	8	16	0
28-31	19	0	4	3	2	1	4	7	0
32-33	13	0	3	4	1	1	3	4	0
34-36	9	0	2	3	1	1	2	3	0
37-38	7	0	2	2	0	1	2	2	0
39-42	6	0	1	2	0	0	1	1	0
**3-4 years**									
	Infection	Perinatal	Congenital anomaly	Injury/external	CNS	Renal/GUI	GI tract	Respiratory	Oral cavity
<28	16	0	4	4	5	1	4	7	1
28-31	9	0	2	4	3	3	3	3	1
32-33	7	0	2	3	1	1	2	3	1
34-36	5	0	1	2	1	1	1	2	1
37-38	4	0	1	2	0	1	1	1	1
39-42	3	0	1	2	0	0	1	1	1
**5-6 years**									
	Infection	Perinatal	Congenital anomaly	Injury/external	CNS	Renal/GUI	GI tract	Respiratory	Oral cavity
<28	8	0	2	3	5	0	3	3	2
28-31	5	0	1	3	3	3	2	2	1
32-33	4	0	1	2	1	1	1	1	1
34-36	3	0	1	2	1	1	1	1	1
37-38	3	0	1	2	0	0	1	1	1
39-42	2	0	1	1	0	0	1	1	1
**7-10 years**									
	Infection	Perinatal	Congenital anomaly	Injury/external	CNS	Renal/GUI	GI tract	Respiratory	Oral cavity
<28	4	0	2	3	5	1	2	2	1
28-31	3	0	1	2	3	3	1	1	1
32-33	3	0	1	2	1	1	1	1	1
34-36	2	0	1	1	0	1	1	1	1
37-38	2	0	1	1	0	1	1	1	1
39-42	1	0	0	1	0	0	1	1	1
